# Copper(II) Trifluoromethanesulfonate as an Efficient, Versatile, and Dual-Purpose Catalyst for the Regioselective Acetylation of l‑Sorbose under Neat and Solvent-Directed Conditions

**DOI:** 10.1021/acsomega.5c11787

**Published:** 2026-01-12

**Authors:** Yu-An Chen, Jasper S. Dumalaog, Cheng-Hsiu Chang, Fu-Chen Liu, Shang-Cheng Hung

**Affiliations:** † 71565 Genomics Research Center, Academia Sinica, Taipei 11529, Taiwan; ‡ Department of Chemistry, National Dong Hwa University, Hualien 97401, Taiwan; § Chemical Biology and Molecular Biophysics Program, Taiwan International Graduate Program (TIGP−CBMB), Academia Sinica, Taipei 11529, Taiwan; ∥ Department of Chemistry, National Tsing Hua University, Hsinchu 30013, Taiwan; ⊥ Department of Chemistry, National Cheng Kung University, Tainan 70101, Taiwan; # Department of Applied Science, National Taitung University, Taitung 95092, Taiwan

## Abstract

**Herein**, we report that copper­(II) trifluoromethanesulfonate [Cu­(OTf)_2_], a stable, versatile, and dual-purpose catalyst, efficiently catalyzed the acetylation of l-sorbose under various conditions following metal trifluoromethanesulfonate screening. Regioselectivity is critically governed by solvents: solvent-free reactions favor the open-chain *keto*-sorbopentaacetate, whereas coordinating solvents direct acetylation toward the cyclic α-l-sorbopyranosyl tetraacetate. These findings underscore the pivotal role of solvents in steering reaction pathways, enabling selective and sustainable synthesis of structurally distinct sugar derivatives while using a minimal amount of catalysts.

## Graphical abstract

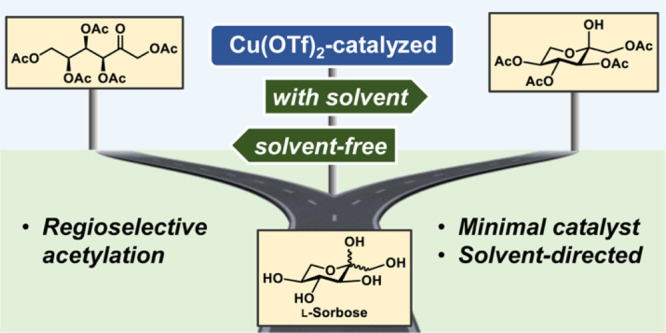

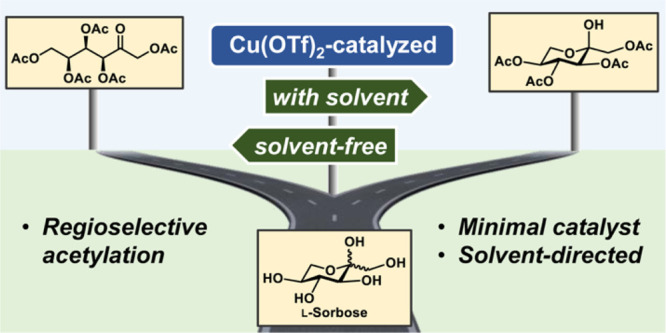

## Introduction

l-Sorbose, a rare monosaccharide obtained by selective oxidation of sorbitol, is a key intermediate in the biosynthesis of ascorbic acid (Vitamin C) in plants and microorganisms.^1^ Although naturally scarce, rare sugars like l-sorbose show significant promise in the food, pharmaceutical, cosmetics, and nutrition industries due to their unique biological properties.^2,3^ l-Sorbose also plays a role in carbohydrate-mediated gene regulation in lactic acid bacteria^4^ and has shown antitumor potential by disrupting glucose metabolism, making it a growing focus in biomedical research.^5^

l-Sorbose exists in equilibrium between open-chain, pyranose, and furanose forms, with the α-pyranose form predominating in aqueous solution (87–98% between 27 and 85 °C),^6^ driven by the anomeric effect. The equatorial positions of most hydroxy groups in the pyranose form contributes to their thermodynamic stability. One particularly important carbohydrate modification is acetylation, which involves the installation of acetyl (Ac) groups to hydroxyl functionalities. Acetylated sugars are considered one of the most prominent glycosyl donors in carbohydrate synthesis, owing to their straightforward preparation, broad availability, and robust chemical stability.^7^ Acetylation modulates sugar recognition, transport, and metabolism.^8^ Acetylated sugars are widely used as glycosyl-donor precursors^9^ and, in glycan labeling, per-*O*-acetylation enhances uptake, with esterases removing acetyl groups intracellularly.^10,11^ Despite its synthetic relevance, l-sorbose acetylation has been the subject of relatively few investigations. The first acetylation of l-sorbose was reported in 1933 by Arragon^12^ and Schlubach^13^ using 14 mol% ZnCl_2_ as Lewis acid catalyst, affording the linear *keto*-l-sorbose 1,3,4,5,6-pentaacetate **2** under neat conditions with excess acetic anhydride (Ac_2_O, Scheme 1). Initially, the Hudson and Brauns acetylation method^14^ employing acetic anhydride and sulfuric acid was applied, but l-sorbose underwent decomposition. Improved yields were obtained when ZnCl_2_ was used as the catalyst. Schlubach^15^ later synthesized the α-l-sorbopyranosyl 1,3,4,5-tetraacetate **3** by using excess Ac_2_O, and pyridine as both base and solvent. Further acetylation with Ac_2_O in the presence of sodium acetate (NaOAc) exclusively afforded the α-l-sorbopyranosyl 1,2,3,4,5-pentaacetate **4α** in 35% yield. Tangara et al.^16^ improved the yield of **3** to 84% using acetyl chloride (AcCl) in pyridine. These acetates serve as versatile precursors^17–19^ but typically require excess reagents and high catalyst loadings. While these methods generate the desired products, a major concern is the difficulty in completely removing pyridine after workup. Furthermore, AcCl is not only sensitive to water but also potentially hazardous, as the reaction can produce corrosive HCl gas. Herein, we report an efficient and regioselective acetylation of l-sorbose catalyzed by metal trifluoromethanesulfonate, with neat and solvent-directed conditions governing regioselectivity. The efficiency of this transformation lies in its low catalyst loading, minimal use of excess reagents, and scalability, offering a practical route for large-scale synthesis. Furthermore, the paper presents the first comprehensive structural characterization of the synthesized sorbose acetates, verifying their structures and confirming the regioselective outcomes.

**Scheme 1:**
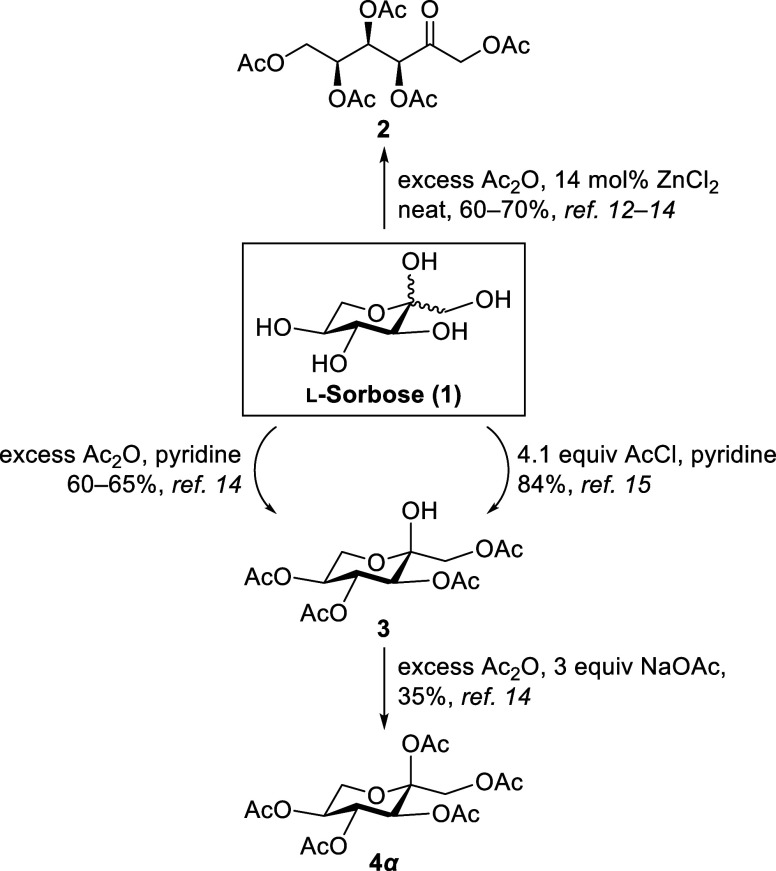
Reported Strategies for the Acetylation of l-Sorbose

## Results and Discussion

Thirteen metal trifluoromethanesulfonates/triflates [M­(OTf)_*n*_] were initially screened under solvent-free conditions to assess their catalytic performance in the acetylation of l-sorbose using 9.5 equiv of Ac_2_O and 1 mol% catalyst at 0 °C to room temperature (Table 1). Mg­(OTf)_2_ (entry 1), an alkaline-earth Lewis acid, was first employed but no product was observed even after 26 h. Similarly, AgOTf (entry 2), a coinage metal triflate, did not yield any product after 26 h. Using Zn­(OTf)_2_ (entry 3) for 7 h afforded **2** in 44% yield, **3** in 3% yield, and a 17% combined yield of inseparable **4α**/**4β** isomers. All yields refer to isolated products from column chromatography. A post-transition metal triflate, Ga­(OTf)_3_ (entry 4), quickly afforded **2** in 38% yield, **3** in 4%, and **4α**+**4β** in 20% yield after 0.5 h.

**Table 1:**
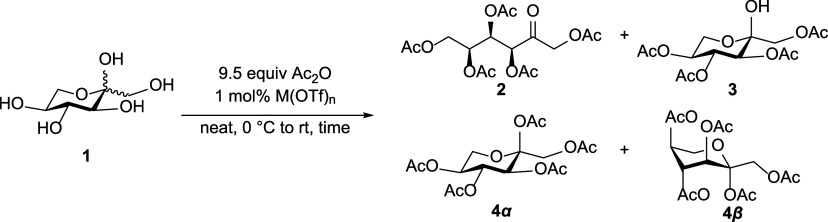
Solvent-Free Acetylation of l-Sorbose under Neat Conditions using Various Metal Trifluoromethanesulfonate Catalysts

			yield (%)		
entry	M(OTf)_n_	time (h)	**2**	**3**	**4α**+**4β** ^a^
1	Mg(OTf)_2_	26	0	0	0
2	AgOTf	26	0	0	0
3	Zn(OTf)_2_	7	44	3	17
4	Ga(OTf)_3_	0.5	38	4	20
5	La(OTf)_3_	24	46	23	17
6	Pr(OTf)_3_	22	45	12	16
7	Nd(OTf)_3_	26	54	16	17
8	Eu(OTf)_3_	26	57	8	19
9	Gd(OTf)_3_	19	57	4	20
10	Dy(OTf)_3_	11	47	3	25
11	Yb(OTf)_3_	17	52	3	27
12	Sc(OTf)_3_	2	46	3	28
13	Cu(OTf)_2_	3	60	6	21

a Reported as a combined yield due to inseparable isomers.

Lanthanide triflates (entries 5–11) showed moderate activity. La­(OTf)_3_ (entry 5), Pr­(OTf)_3_ (entry 6), and Nd­(OTf)_3_ (entry 7) gave **2** in 45–54%, **3** in 12–23%, and **4α+4β** in ∼16–17% yields over 22–26 h. Eu­(OTf)_3_ (entry 8) and Gd­(OTf)_3_ (entry 9) afforded **2** in 57% with lower **3** (4–8%) and 19–20% **4α+4β** (19–26 h). Dy­(OTf)_3_ (entry 10) and Yb­(OTf)_3_ (entry 11) yielded **2** in 47–52%, **3** in 3%, and higher **4α+4β** (25–27%). Sc­(OTf)_3_ (entry 12), an early transition-metal triflate previously used in our group’s per-*O*-acetylation studies on hexoses,^20^ produced **2** (46%), **3** (3%), and **4α**+**4β** (28%) within 2 h. Finally, Cu­(OTf)_2_ (entry 13), a late transition-metal triflate which was shown in our prior work to be also effective in the per-acetylation of hexoses,^21^ efficiently afforded **2** in 60%, **3** in 6%, and **4α**+**4β** in 21% yield after 3 h. ^1^H NMR of the crude product indicated a **4α**/**4β** ratio of 1.5/1.0. Owing to its superior efficiency, Cu­(OTf)_2_ was chosen for subsequent studies, with its stability, cost-effectiveness, low toxicity, and strong Lewis acidity highlighting its dual role as a versatile transition-metal catalyst.^22,23^

For structural analysis, pure sorbose acetates **2**–**4** were recrystallized and characterized. X-ray structures of **2** (CCDC 2449038, Figure 1A), **3** (CCDC 2449039, Figure 1B), and **4α** (CCDC 2449041, Figure 1C) confirmed their molecular structures and matched with their corresponding NMR data. Crystals of **2** and **3** were readily obtained, whereas only the α-isomer whereas only the α-isomer (**4α**) could be isolated from the **4α+4β** mixture after recrystallization.

**Figure 1:**
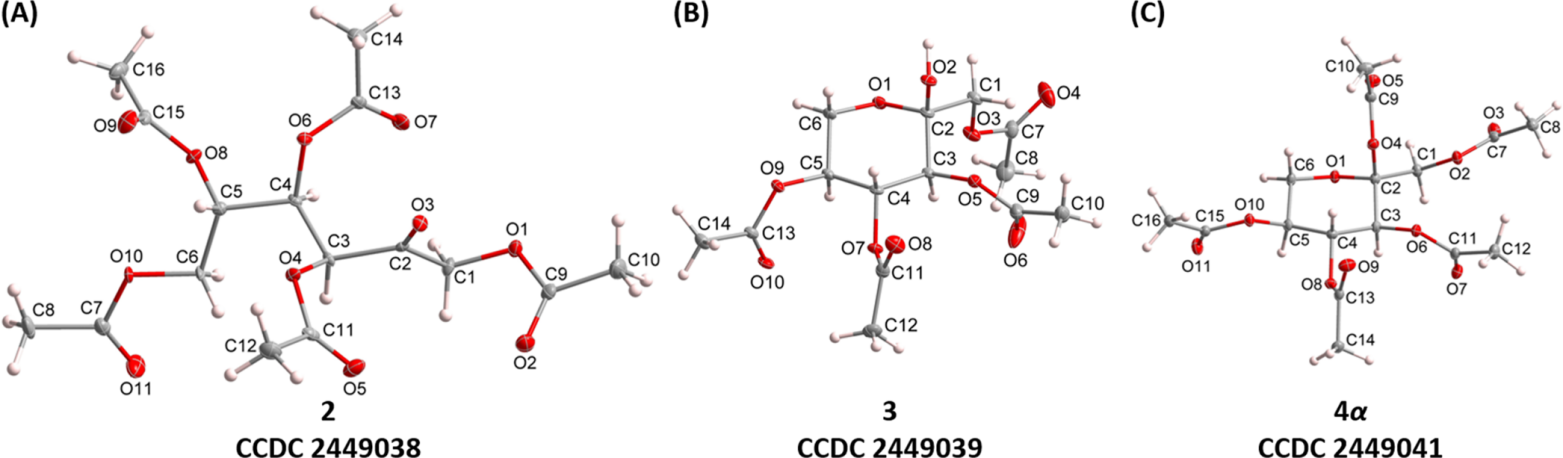
X-ray single crystal structure of l-sorbose acetates (A) **2**; (B) **3**; and (C) **4α**.

The structure of **2** was confirmed as open-chain by *keto* C=O at C2–O3 in the X-ray structure (Figure 1A), resonating at δ = 197.1 ppm in the ^13^C NMR spectrum. This *xylo*-configured acyclic sugar adopts a bent, sickle-like conformation rather than a fully zigzag chain to minimize 1,3-*syn* interactions between the acetate groups at C3 and C5.^24^ This is further supported by the torsion angles measured from the X-ray structure using Mercury,^25^ with C2–C3–C4–C5 ≈ 180° and C3–C4–C5–C6 ≈ 60°, reflecting a 120° rotation along the C–C bond to minimize these interactions.^26^ Compound **2** also adopts a *gauche* orientation between C4 and C5.^27^ The ^1^H NMR coupling constants (^3^ *J*_H3–H4_ = 3.7 Hz and ^3^ *J*_H4–H5_ = 5.9 Hz), and corresponding torsion angles (H3–C3–C4–H4 = 50.2° and H4–C4–C5–H5 = 170.5°), further corroborate this bent conformation.

To confirm the cyclic structure of **3**, Cremer–Pople analysis yielded *Q* = 0.583 Å, θ = 175.5°, and φ = 106.6°, where *Q* is the puckering amplitude, θ defines the puckering type, and φ the phase. The θ value near 180° indicates that the pyranose ring of **3** adopts a ^1^ *C*_4_ chair conformation (Figure 1B).^28^ Consistently, l-sorbopyranose rings in the ^1^ *C*_4_ chair conformation typically display ^3^ *J*_H3–H4_ ≈ 10 Hz and ^3^ *J*_H4–H5_ ≈ 9 Hz.^29^ For **3**, the observed coupling constants of 9.8 Hz for both ^3^ *J*_H3–H4_ and ^3^ *J*_H4–H5_ agree with the X-ray assignment, further supported by the torsion angles of H3–C3–C4–H4 = 170.1° and H4–C4–C5–H5 = 173.6°.

The anomeric configuration of **3** was confirmed by two-dimensional nuclear Overhauser effect spectroscopy (2D NOESY). Since l-sorbopyranose lacks an anomeric proton, the usual one-bond ^1^ *J*_C1–H1_ coupling cannot distinguish α- and β-isomers. NOESY correlations between the anomeric hydroxy proton and H1, H4, and H6 established its axial orientation, confirming the α-configuration, further supported by W-coupling with H3 in 2D COSY due to hydrogen bonding with the C1 acetyl carbonyl (Figure 2A). Cremer–Pople parameters for **4α** (Q = 0.550 Å, θ = 172.1°, φ = 234.8°) likewise indicate a ^1^ *C*_4_ conformation (Figure 1C). NMR analysis confirmed this assignment, with ^3^ *J*_H3–H4_ and ^3^ *J*_H4–H5_ both at 9.8 Hz, matching the torsion angles H3–C3–C4–H4 = 168.6° and H4–C4–C5–H5 = 161.0°. In contrast, elucidation of **4β** was challenging due to its inseparability from the **4α/4β** mixture. ^1^H NMR showed two sets of resonances, and NOESY correlations were nearly identical for both isomers, precluding straightforward differentiation.

**Figure 2:**
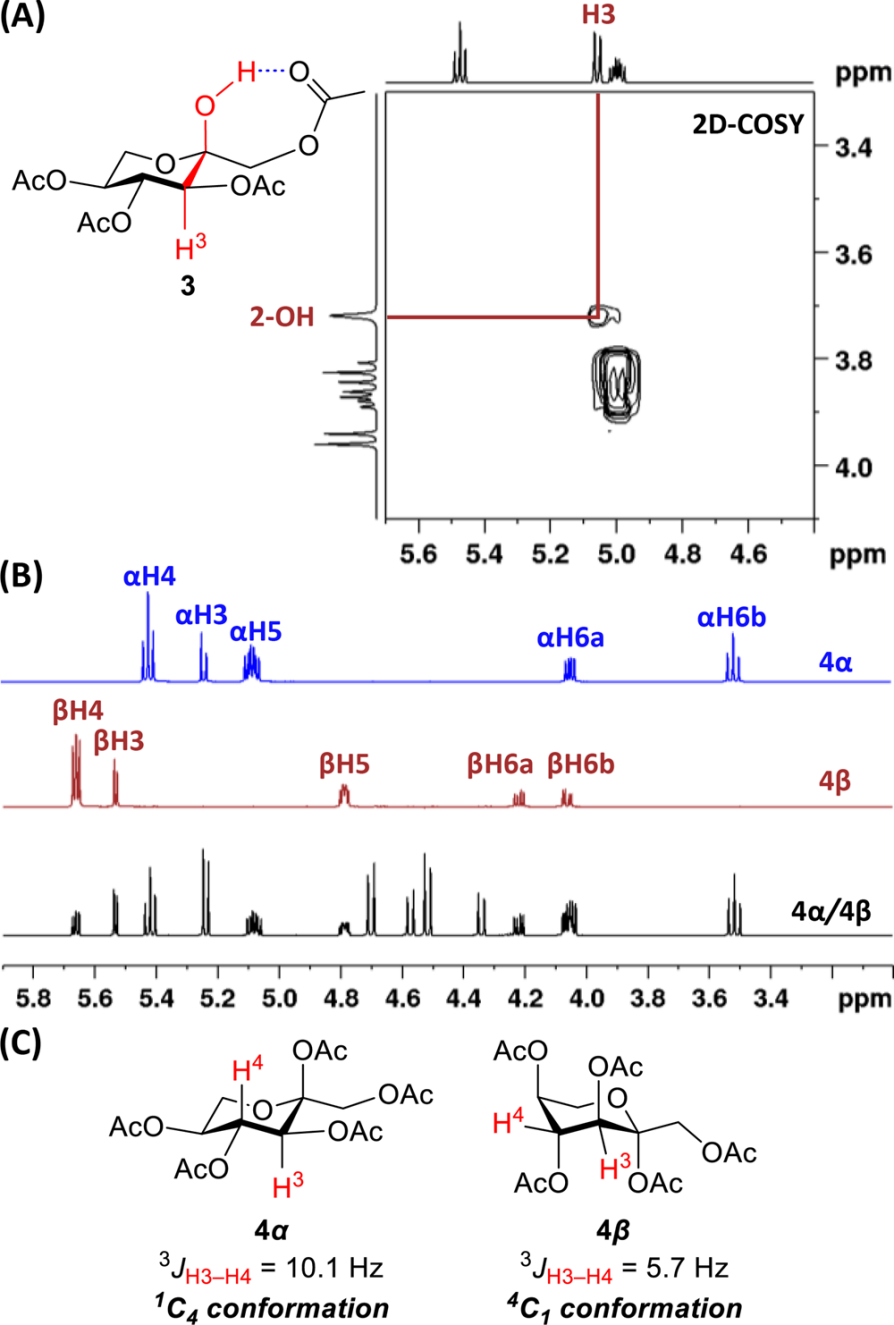
(A) 2D COSY of **3**, showing the W-coupling between H3 and OH; (B) 1D TOCSY of the **4α/4β** mixture; and (C) coupling constants of **4α** and **4β** distinguishing their chair conformations.

To resolve the isomers, ^13^C NMR spectrum of the mixture was examined. Previous studies have established that the β-anomeric carbon of l-sorbopyranose resonates further downfield compared to the α-isomer.^30–32^ In agreement, the **4α/4β** mixture displayed two distinct anomeric carbon signals: δ_C_ = 104.5 ppm (**4β**), and δ_C_ = 101.8 ppm (**4α**). The latter matched the anomeric carbon signal of crystalline **4α**, validating the assignment.

Selective one-dimensional total correlation spectroscopy (1D TOCSY) was used to deconvolute the overlapping ^1^H spectra. Excitation of the ^1^H nuclei of H3 (doublet resonance) from each isomer, the spectra resolved into distinct proton patterns for **4α** and **4β** (Figure 2B). Assignments were supported by 2D correlation spectroscopy (COSY), confirming intraring proton connectivity (excluding exocyclic H1). The 1D TOCSY of **4α** also matched the proton pattern of crystalline **4α**, further confirming the assignments.

Conformational analysis revealed that **4α** retains the ^1^ *C*_4_ chair, consistent with ^3^ *J*_H3–H4_ = 10.1 Hz and ^3^ *J*_H4–H5_ = 9.8 Hz, which verified the *trans*-diaxial arrangement of H3, H4, and H5. On the other hand, **4β** exhibited reduced coupling constants (^3^ *J*_H3–H4_ = 5.7 Hz, ^3^ *J*_H4–H5_ = 7.0 Hz), indicative of a possible shift to the ^4^ *C*_1_ conformation (Figure 2C). This trend parallels Paulsen’s classic observation of a drop in ^3^ *J*_H3–H4_ (9.0–4.0 Hz) upon Walden inversion of tetra-*O*-benzoyl-α-l-sorbopyranose to its β-anomer.^33^ Notably, in **4β**, all four acetyl groups, including the anomeric substituent, occupy axial positions, making this conformational switch particularly striking. The axial preference of the electronegative acetyl group at C2 is stabilized by the anomeric effect.^34^ Attempts to recrystallize pure **4β** from the isomeric mixture were unsuccessful. Isolation of crystalline **4β** would conclusively confirm the proposed conformational change and provide insight into the key torsion angles. Melting points, mass spectrometry, and specific optical rotation of the sorbose acetates matched reported data,^35^ confirming their structure and purity.

To determine the optimal amount of Ac_2_O in the Cu­(OTf)_2_-catalyzed solvent-free acetylation of l-sorbose, reactions were performed with varying equivalents of Ac_2_O, 1 mol% Cu­(OTf)_2_, and a fixed reaction time of 3 h (Table 2, entries 1–5). Based on the metal triflate screening in Table 1, Cu­(OTf)_2_ with 9.5 equiv of Ac_2_O was selected as the starting point (entry 1). Using 5.1 equiv of Ac_2_O (entry 2), pentaacetate **2** was the major product (31%), with minor **3** (13%) and combined **4α**+**4β** (10%). Increasing Ac_2_O to 6.1 equiv (entry 3) improved **2** to 41%, while 7.1 equiv (entry 4) and 8.1 equiv (entry 5) further increased **2** to 46 and 56%, respectively, with minor **3**, **4α**, and **4β** in varying proportions. Under the optimal condition (entry 1), the **4α**/**4β** ratio was 1.5/1.0 by ^1^H NMR. These results indicate that 9.5 equiv of Ac_2_O is required to achieve the highest yield of the open-chain pentaacetate **2** under neat conditions.

**Table 2:**
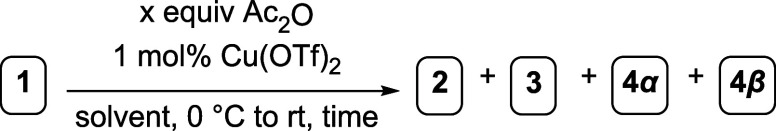
Cu­(OTf)_2_-Catalyzed Acetylation of l-Sorbose (1) with Different Amounts of Ac_2_O

				yield (%)		
entry	*x*	solvent	time (h)	**2**	**3**	**4α**+**4β** ^a^
1	9.5	none	3	60	6	21
2	5.1	none	3	31	13	10
3	6.1	none	3	41	17	14
4	7.1	none	3	46	14	16
5	8.1	none	3	56	11	21
6	4.1	THF	18	2	72	0
7	5.1	THF	18	6	65	3
8	6.1	THF	18	10	58	3
9	7.1	THF	18	13	51	9
10	8.1	THF	18	18	46	11
11	9.5	THF	18	23	44	12

a Reported as a combined yield due to inseparable isomers.

In contrast, reactions in tetrahydrofuran (THF) with 1 mol% Cu­(OTf)_2_ and 18 h reaction time (Table 2, entries 6–11) required a longer reaction time to reach product completion after the solvent was introduced. However, these reactions resulted in a significantly different product distribution compared to the neat conditions. Starting with 4.1 equiv of Ac_2_O (entry 6), the reaction afforded the α-l-sorbopyranosyl 1,3,4,5-tetraacetate **3** as the major product (72%), while **2** was obtained in only 2%, and **4α**+**4β** was undetected. This marked shift in selectivity indicates that solvent presence strongly influences the reaction pathway. As the amount of Ac_2_O was increased to 5.1, 6.1, 7.1, 8.1, and 9.5 equiv (entries 7–11), the yield of product **3** gradually decreased to 65, 58, 51, 46, and 44%, respectively, with various minor products, including **2** and **4α**+**4β**, appearing in lower yields. These results suggest that 4.1 equiv of Ac_2_O is optimal for regioselectively generating **3** in THF, while excess Ac_2_O reduces both yield and regioselectivity.

To further optimize the solvent-free acetylation of l-sorbose, Cu­(OTf)_2_ catalyst loading was varied from 1 to 0.2 mol%, with reaction times adjusted accordingly until completion (Table 3). All reactions were conducted from 0 °C to room temperature. At 1 mol% catalytic amount (entry 1), the reaction proceeded for 3 h yielding **2** in 60%, with minor products **3** (6%), and **4α**+**4β** (21%). Reducing the catalyst to 0.5 mol% and with a reaction time of 4 h (entry 2) maintained the same yield of **2** (60%). Further lowering to 0.4 mol% (entry 3, 5 h) slightly improved **2** to 64%, representing the optimal condition. At this point, the ratio of **4α**/**4β** was found to be 1.4/1.0 by ^1^H NMR. Lower catalytic amounts of 0.3, 0.25, and 0.2% (entries 4–6) required 24 h and gave product **2** in 54, 55, and 49% yields, respectively. Under the optimized conditions, the reaction was successfully scaled up to 20 g, yielding 61% of **2**. These results identify 0.4 mol% Cu­(OTf)_2_ as optimal, demonstrating efficient acetylation with minimal catalyst and highlighting the method’s practicality and sustainability for broader synthetic applications.

**Table 3:**
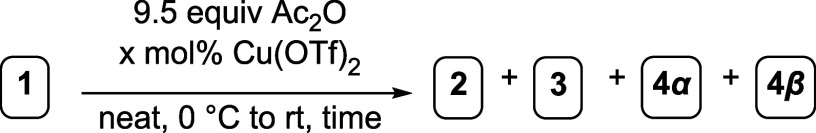
Cu­(OTf)_2_-Catalyzed Solvent-Free Acetylation of l-Sorbose (1) with Excess Ac_2_O under Varying Catalyst Loading

			yield (%)		
entry	x	time (h)	**2**	**3**	**4α**+**4β** ^a^
1	1	3	60	6	21
2	0.5	4	60	0	23
3	0.4	5	64	0	24
4	0.3	24	54	3	21
5	0.25	24	55	5	21
6	0.2	24	49	12	20

a Reported as a combined yield due to inseparable isomers.

The influence of solvent on Cu­(OTf)_2_-catalyzed l-sorbose acetylation with 4.1 equiv Ac_2_O was examined under standardized conditions (0.4 mol% Cu­(OTf)_2_, from 0 °C to room temperature, Table 4). No reaction occurred in acetonitrile (CH_3_CN, entry 2). Reactions in THF (entry 1) and diethyl ether (Et_2_O, entry 3) afforded **3** in lower yields of 19 and 41%, respectively. Noncoordinating solvents such as dichloromethane (CH_2_Cl_2_, entry 4) and chloroform (CHCl_3_, entry 5) gave mixed product distributions. Among the solvents tested, 1,4-dioxane (entry 6) and diglyme (entry 7) gave the highest conversions to the desired major product, affording **3** in 67 and 68% yield, respectively, along with minor amounts of **2** and **4α**+**4β**. These findings highlight the pivotal influence of solvent on regioselectivity, with 1,4-dioxane and diglyme promoting the selective formation of **3** under the optimized conditions. The optimized dioxane-directed acetylation was then applied on a 20 g scale, demonstrating scalability and yielding compound **3** in 60%. Coordinating solvents can significantly enhance regioselectivity by modulating Lewis acid catalyst activity and forming stabilizing interactions with hydroxyl groups, which in turn alter substrate conformation and reactivity.^36,37^ Their ability to coordinate with Cu­(OTf)_2_, as in THF, 1,4-dioxane, and diglyme, likely explains the solvent-dependent regioselectivity of the acetylation.

**Table 4:**
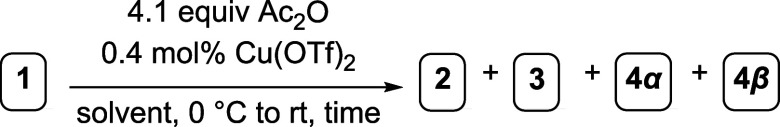
Cu­(OTf)_2_-Catalyzed Acetylation of l-Sorbose (1) with Stoichiometric Ac_2_O in Different Solvents

			yield (%)		
entry	solvent	time (h)	**2**	**3**	**4α**+**4β** ^a^
1	THF	27	3	19	0
2	CH_3_CN	24	0	0	0
3	Et_2_O	24	21	41	2
4	CH_2_Cl_2_	24	18	14	28
5	CHCl_3_	24	43	13	16
6	1,4-Dioxane	6	4	67	0
7	Diglyme	4.5	4	68	0

a Reported as a combined yield due to inseparable isomers.

A pure compound **4α**, was efficiently obtained via the direct acetylation of **3**. As shown in Table 5, 4-(dimethylamino)­pyridine (DMAP)-catalyzed acetylation of the tertiary axial alcohol in **3** with 1.5 equiv of Ac_2_O and 3 equiv of base in CH_2_Cl_2_ at room temperature afforded **4α**. Using *N*,*N*,*N*′,*N*′-tetramethylethylenediamine (TEMED, entry 1) afforded **4α** in 71% yield after 16 h, while triethylamine (Et_3_N, entry 2) gave a higher 83% yield within 6 h, with no detectable formation of **4β** in either case. Full characterization of the recrystallized pentaacetate **4α**, including NMR spectroscopy and X-ray crystallography, confirmed the expected structure, ring conformation, and high purity. This approach underscores the efficiency of DMAP-catalyzed acetylation in directly yielding pure **4α**. In contrast, Cu­(OTf)_2_-catalyzed acetylation of l-sorbose with excess Ac_2_O produced a mixture of **4α** and **4β**, while direct acetylation of **3** under the same conditions gave a mixture of **2**, **4α**, and **4β**.

**Table 5:**
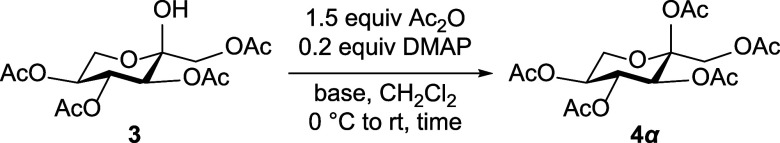
Direct Acetylation of 3 to Acquire the Pure 4α Isomer

entry	base (equiv)	time (h)	yield (%)
1	TEMED (3.0)	16	71
2	Et_3_N (3.0)	6	83

To probe the formation of linear pentaacetate **2** under neat conditions, NMR analysis was carried out. Upon addition of Cu­(OTf)_2_, the tetraacetate intermediate **3** formed rapidly, with H3 (5.06 ppm) and H5 (5.00 ppm) signals prominent at the start (Figure 3). These signals gradually disappeared as the reaction progressed, consistent with TLC observations. Intermediate **3** subsequently underwent acetylation to afford pentaacetates **2** as the major product and minor **4α+4β**, confirming **3** is a key intermediate capable of either ring opening to form **2** or direct acetylation to **4α+4β**. As shown in Scheme 2A, the proposed mechanism involves Cu­(OTf)_2_ catalysis, activating Ac_2_O toward reaction with l-sorbose, with the catalyst regenerated for subsequent cycles. The formation of **3**, as indicated by NMR, is likely due to the reduced reactivity of the tertiary axial alcohol compared to the other −OH groups in l-sorbose. A similar phenomenon has been observed with D-fructose, where a tetraacetate intermediate forms first, analogous to the formation of **3**, before proceeding to the pentaacetate during acetylation with ZnCl_2_.^38^ The Lewis acid catalyst facilitates the ring opening of the pyranose ring, followed by acetylation to yield the pentaacetate products **2**, **4α**+**4β**.

**Figure 3:**
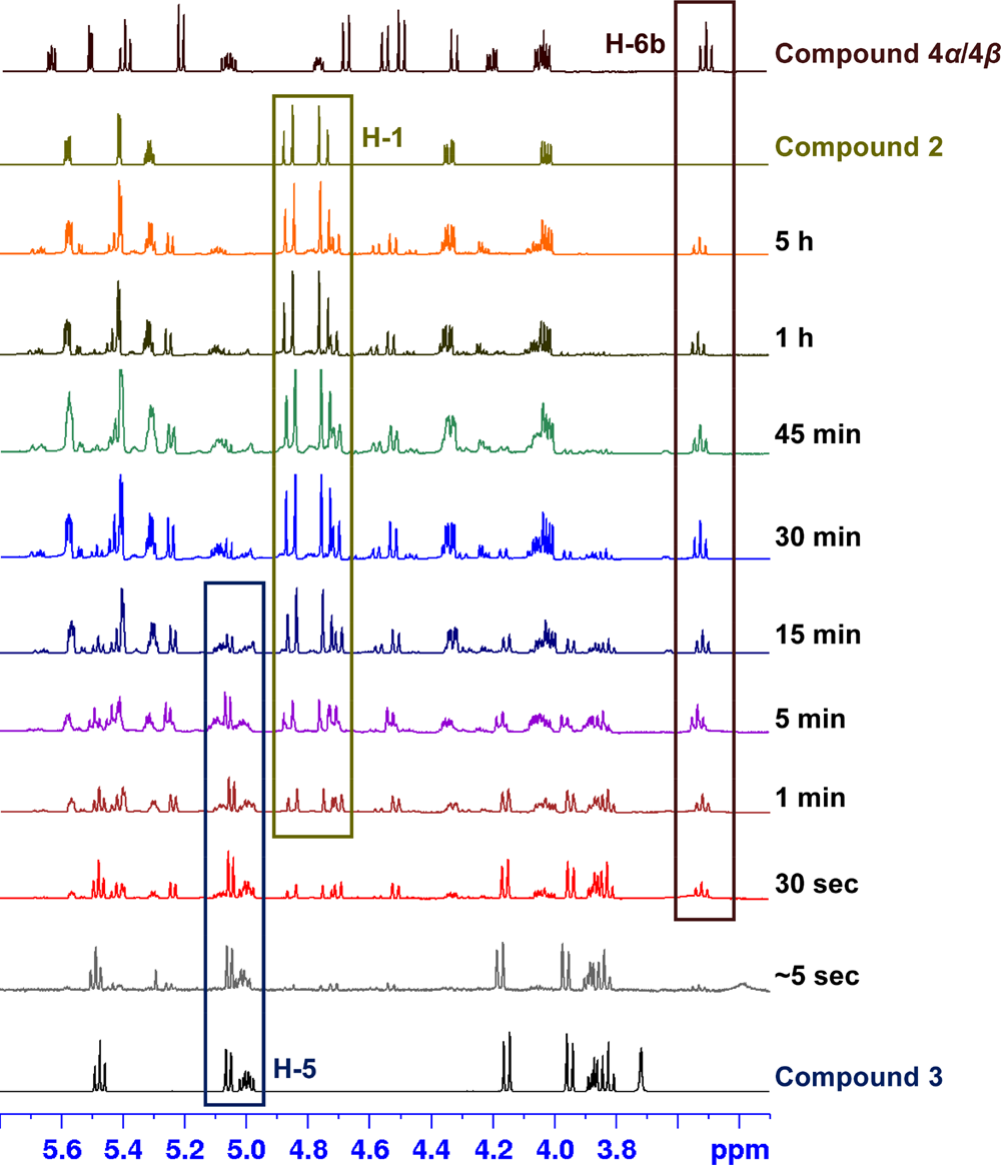
NMR monitoring of l-sorbose solvent-free acetylation.

**Scheme 2:**
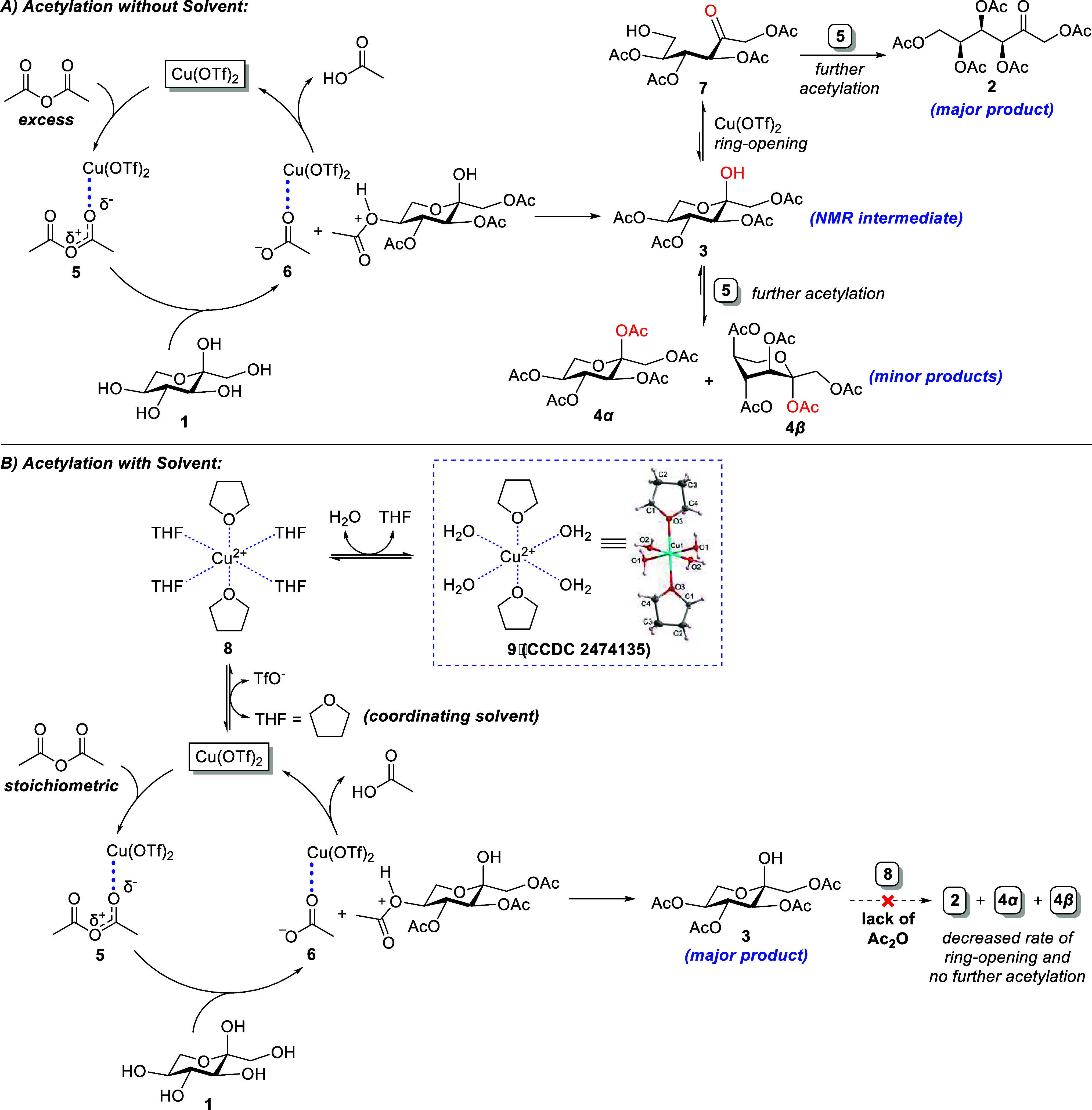
Proposed Reaction Mechanisms for the Cu­(OTf)_2_-Catalyzed Acetylation of l-sorbose (A) without or (B) with the Solvent

In a complementary aspect, we also explore the mechanistic details of Cu­(OTf)_2_-catalyzed acetylation of l-sorbose in coordinating solvents, as illustrated in Scheme 2B. Upon acetylation, tetraacetate **3** is formed, and the catalyst further interacts with the solvent. Notably, a single crystal of the Cu­(OTf)_2_–THF complex (**9**, CCDC 2474135) was successfully isolated, providing critical insights into the proposed mechanism in THF. In coordinating solvents, THF displaces the triflate ligands of the catalyst, resulting in the formation of an octahedral Cu–THF complex (**8**), which equilibrates with crystal water to yield **9**. This was confirmed by both the ORTEP diagram of complex **9** (Figure S4) and NMR spectra (Figures S33 and S34), which show the resonance associated with THF. The crystallographic evidence of the catalyst–solvent complex offers an explanation for the reduced Lewis acidity of the catalyst, which subsequently diminishes the rate of ring opening and further acetylation, thereby preventing the formation of **2**, **4α**, and **4β**. These findings suggest that regioselectivity is significantly influenced by the coordinating properties of the solvent, which affect the catalyst’s ability to open the pyranose ring.

## Conclusions

In summary, we have established a highly efficient and regioselective acetylation of l-sorbose using only 0.4 mol% Cu­(OTf)_2_, which is a versatile and dual-purpose catalyst. The regioselectivity of the reaction was shown to be solvent-dependent, favoring open-chain peracetylation under neat conditions and cyclic tetraacetate formation in coordinating solvents. The method demonstrates high scalability and operational simplicity, making it suitable for large-scale or industrial applications. Overall, this work provides a sustainable platform for the acetylation of rare sugars such as l-sorbose with potential applications in carbo-hydrate chemistry and beyond.

## Experimental Section

### General Methods

All reactions requiring anhydrous conditions were performed under a nitrogen atmosphere in flame-dried glassware. Solvents such as CH_2_Cl_2_, CH_3_CN, and THF were distilled through a purification system with activated Al_2_O_3_. The commercial reagents were used without further purification, unless otherwise stated. TLC analysis was performed on Silica Gel 60G F_254_ glass plates (0.25 mm, E. Merck). TLC plates were visualized by exposure to UV light (UV-254 nm), and the detection was done by spraying with a solution of Hanessian’s stain containing Ce­(NH_4_)_2_(NO_3_)_6_, (NH_4_)_6_Mo_7_O_24_, and H_2_SO_4_ in water and subsequent heating on a hot plate. Flash column chromatography was done on Silica Gel 60 (230–400 mesh, E. Merck). Specific rotations were taken at ambient temperature using HORIBA Sepa-300 Polarimeter at 589 nm (sodium D line) and reported in 10^–1^·deg·cm^2^·g^–1^ where the sample concentrations are in g·dL^–1^. The measurements of the IR spectra were done on KBr plates using PerkinElmer Spectrum 100 FT-IR Spectrometer. 1D and 2D NMR spectra were acquired from Bruker Avance III 600 MHz spectrometer at ambient temperature. Data were recorded as follows: chemical shift in ppm from the solvent resonance employed as the internal standard (CDCl_3_ at 7.26 ppm), multiplicity (s = singlet, d = doublet; t = triplet; q = quartet; m = multiplet; bs = broad singlet), coupling constant in Hz, and integration. ^13^C NMR spectra were measured on a 150 MHz spectrometer. Chemical shifts were recorded in ppm from the solvent resonance employed as the internal standard (CDCl_3_ at 77.0 ppm). X-ray crystallography data for single crystals were collected using a Bruker D8 Venture X-ray diffractometer. Melting points were determined with a Fargo MP-2D melting point apparatus. Mass spectra were obtained with ESI Finnigan LCQ mass spectrometer (Thermo Finnigan).

### Acetylation in Solvent-Free Conditions (**Method A**)

Dried l-Sorbose **1** (500 mg, 2.78 mmol) was treated with acetic anhydride (Ac_2_O, 2.50 mL, 9.53 mmol, 9.5 equiv) and stirred for 10 min. The reaction flask was allowed to stir at 0 °C, then, freshly dried copper­(II) trifluoromethanesulfonate (Cu­(OTf)_2_, 0.4 mol%) was added to the reaction mixture. After 15 min, the reaction mixture was gradually warmed to room temperature and was further stirred under nitrogen atmosphere for 5 h. The complete consumption of the starting material was confirmed by TLC analysis (ethyl acetate/hexane = 1/1). The reaction mixture was then diluted with EtOAc and saturated solution of NaHCO_3(aq)_ was added. The crude product was extracted with EtOAc three times. The combined organic layers were washed with brine, dried over anhydrous MgSO_4_, filtered, and concentrated *in vacuo*. Compound **2** was first precipitated as a white solid using cold ethanol, and the remaining crude products were purified by flash column chromatography (ethyl acetate/hexane = 1/3) on silica gel to afford the acetylated products **2**, **3**, **4α**, and **4β**.

### Acetylation in Solvent-Directed Conditions (**Method B**)

A solution of dried l-sorbose **1** (500 mg, 2.78 mmol) in 1,4-dioxane (5.00 mL) was stirred at room temperature for 10 min. Ac_2_O (1.08 mL, 11.4 mmol, 4.1 equiv) was then added, and the mixture was stirred for an additional 10 min. The reaction flask was cooled in an ice bath at 0 °C. Subsequently, freshly dried 0.4 mol% Cu­(OTf)_2_ was added to the reaction mixture. The ice bath was removed, then the reaction was allowed to stir under an N_2_ atmosphere until complete consumption of the starting material was confirmed by TLC analysis (ethyl acetate/hexane = 1/1). The reaction mixture was diluted with EtOAc and quenched with saturated aqueous NaHCO_3_. The crude product was extracted with EtOAc three times. The combined organic layers were washed with brine, dried over anhydrous MgSO_4_, filtered, and concentrated *in vacuo*. The crude product of purified by flash column chromatography (ethyl acetate/hexane = 1/3) on silica gel to get the acetylated compounds **2**, **3**, **4α**, and **4β**.

### 1,3,4,5,6-Penta-*O*-acetyl-*keto*-l-sorbose (**2**)

**Method A** furnished compound **2** (694 mg, 64%) as a white solid, whereas **Method B** produced the same compound **2** (44 mg, 4%) as a white solid. Single crystals (CCDC 2449038) suitable for X-ray analysis were obtained by recrystallization from ethyl acetate. *R*_f_ = 0.33 (ethyl acetate/hexane = 1/1); mp 101–102 °C; [α]_D_^25^ + 10.9 (*c* = 1.0, CHCl_3_); IR (thin film): ν 2943, 1748, 1432, 1374, 1220, 1048 cm^–1^; ^1^H NMR (600 MHz, CDCl_3_) δ_H_ 5.56 (dd, *J* = 3.6, 5.9 Hz, 1H, H-4), 5.39 (d, *J* = 3.7 Hz, 1H, H-3), 5.29 (td, *J* = 4.5, 5.8 Hz, 1H, H-5), 4.84 (d, *J* = 17.2 Hz, 1H, H-1a), 4.72 (d, *J* = 17.1 Hz, 1H, H-1b), 4.31 (dd, *J* = 4.5, 12.1 Hz, 1H, H-6a), 4.00 (dd, *J* = 12.1, 5.7 Hz, 1H, H-6b), 2.18 (s, 3H, Ac CH_3_), 2.12 (s, 3H, Ac CH_3_), 2.09 (s, 3H, Ac CH_3_), 2.03 (s, 3H, Ac CH_3_), 2.03 (s, 3H, Ac CH_3_); ^13^C NMR (150 MHz, CDCl_3_) δ_C_ 197.1 (*keto* C=O), 170.3 (Ac C=O), 169.64 (Ac C=O), 169.59 (Ac C=O), 169.57 (Ac C=O), 169.4 (Ac C=O), 73.9 (CH, C-3), 68.93 (CH, C-5), 68.88 (CH, C-4), 66.4 (CH_2_, C-1), 61.5 (CH_2_, C-6), 20.54 (Ac CH_3_), 20.48 (Ac CH_3_), 20.4 (Ac CH_3_), 20.3 (Ac CH_3_), 20.2 (Ac CH_3_); HRMS–ESI (*m*/*z*): [M+NH_4_]^+^ calcd for C_12_H_16_O_8_NH_4_^+^, 408.1500; found, 408.1505.

### 1,3,4,5-Tetra-*O*-acetyl-α-l-sorbopyranose (**3**)

**Method A** did not produce compound **3**, whereas **Method B** yielded compound **3** (645 mg, 67%) as a cloudy white syrup. Single crystals (CCDC 2449039) suitable for X-ray analysis were obtained by recrystallization from dichloromethane/hexane. *R*_f_ = 0.22 (ethyl acetate/hexane = 1/1); mp 105–106 °C; [α]_D_^25^ – 9.5 (*c* = 1.0, CHCl_3_); IR (thin film): ν 3443, 2958, 1737, 1432, 1370, 1235, 1107, 1057 cm^–1^; ^1^H NMR (600 MHz, CDCl_3_) δ_H_ 5.47 (t, *J* = 9.8 Hz, 1H, H-4), 5.06 (d, *J* = 9.9 Hz, 1H, H-3), 5.00 (ddd, *J* = 6.1, 9.6, 10.6 Hz, 1H, H-5), 4.15 (d, *J* = 11.8 Hz, 1H, H-1a), 3.95 (d, *J* = 11.8 Hz, 1H, H-1b), 3.87 (dd, *J* = 6.2, 11.0 Hz, 1H, H-6a), 3.27 (t, *J* = 10.8 Hz, 1H, H-6b), 3.72 (s, 1H, 2-OH), 2.10 (s, 3H, Ac CH_3_), 2.06 (s, 3H, Ac CH_3_), 2.02 (s, 3H, Ac CH_3_), 2.00 (s, 3H, Ac CH_3_); ^13^C NMR (150 MHz, CDCl_3_) δ_C_ 170.9 (Ac C=O), 170.2 (Ac C=O), 169.9 (Ac C=O), 169.7 (Ac C=O), 95.6 (C-2), 70.6 (CH, C-4), 70.2 (CH, C-3), 69.1 (CH, C-5), 65.6 (CH_2_, C-1), 59.3 (CH_2_, C-6), 20.7 (Ac CH_3_), 20.64 (Ac CH_3_), 20.62 (Ac CH_3_), 20.5 (Ac CH_3_); HRMS–ESI (*m*/*z*): [M+NH_4_]^+^ calcd for C_14_H_20_O_10_NH_4_^+^ 366.1395; found, 366.1401.

### 1,2,3,4,5-Penta-*O*-acetyl-α/β-l-sorbopyranose (**4α**/**4β**, Mixed Isomers)

**Method A** afforded mixed isomers of compound **4α** and **4β** (255 mg, 24%, α/β = 1.0/1.4) as a cloudy white syrup, whereas **Method B** did not produce these compounds. *R*_f_ = 0.36 (ethyl acetate/hexane = 1/1); [α]_D_^25^ – 37.6 (*c* = 1.0, CHCl_3_); IR (thin film): ν 2973, 1755, 1435, 1371, 1222, 1055 cm^–1^; ^1^H NMR (600 MHz, CDCl_3_) δ_H_ 5.63 (dd, *J* = 5.7, 7.0 Hz, 1H, β H-4), 5.51 (d, *J* = 5.7 Hz, 1H, β H-3), 5.40 (t, *J* = 9.8 Hz, 1H, α H-4), 5.21 (d, *J* = 10.1 Hz, 1H, α H-3), 5.06 (ddd, *J* = 6.1, 9.8, 10.6 Hz, 1H, α H-5), 4.76 (ddd, *J* = 4.5, 5.3, 7.2 Hz, 1H, β H-5), 4.68 (d, *J* = 12.0 Hz, 1H, α H-1a), 4.55 (d, *J* = 11.8 Hz, 1H, β H-1a), 4.49 (d, *J* = 11.9 Hz, 1H, α H-1b), 4.32 (d, *J* = 11.8 Hz, 1H, β H-1b), 4.20 (dd, *J* = 5.5, 12.2 Hz, 1H, β H-6a), 4.04 (dd, *J* = 4.2, 12.1 Hz, 1H, β H-6b), 4.02 (dd, *J* = 6.1, 11.3 Hz, 1H, α H-6a), 3.50 (t, *J* = 11.1 Hz, 1H, α H-6b), 2.16 (s, 3H, Ac CH_3_), 2.06–2.05 (m, 15H, Ac CH_3_), 2.03 (s, 3H, Ac CH_3_), 2.00 (s, 6H, Ac CH_3_), 1.98 (s, 3H, Ac CH_3_); ^13^C NMR (150 MHz, CDCl_3_) δ_C_ 170.4 (Ac C=O), 170.1 (Ac C=O), 169.93 (2 × Ac C=O), 169.85 (Ac C=O), 169.6 (Ac C=O), 169.4 (Ac C=O), 169.3 (Ac C=O), 168.5 (Ac C=O), 167.8 (Ac C=O), 104.5 (C, β C-2), 101.8 (C, α C-2), 77.1 (CH, β C-5), 76.4 (CH, β C-3), 75.7 (CH, β C-4), 70.4 (CH, α C-4), 68.9 (CH, α C-3), 68.3 (CH, α C-5), 64.0 (CH_2_, α C-1), 62.2 (CH_2_, β C-1), 62.1 (CH_2_, β C-6), 61.1 (CH_2_, α C-6), 21.5 (Ac CH_3_), 21.4 (Ac CH_3_), 20.8 (Ac CH_3_), 20.64 (Ac CH_3_), 20.58 (3 × Ac CH_3_), 20.5 (Ac CH_3_), 20.4 (2 × Ac CH_3_); HRMS–ESI (*m*/*z*): [M+NH_4_]^+^ calcd for C_16_H_22_O_11_NH_4_^+^ 408.1500; found, 408.1507.

### 1,2,3,4,5-Penta-*O*-acetyl-α-l-sorbopyranose (**4α**)

In order to obtain pure **4α**, dried compound **3** (130 mg, 0.37 mmol) was dissolved in dry dichloromethane (1.30 mL). The reaction mixture was then allowed to stir at 0 °C. Afterward, Ac_2_O (0.05 mL, 0.53 mmol, 1.5 equiv) was added to the solution, followed by the addition of Et_3_N (0.16 mL, 1.15 mmol, 3 equiv), and DMAP (9.12 mg, 0.07 mmol, 0.2 equiv). The reaction mixture was gradually warmed to room temperature and was continuously stirred under an N_2_ atmosphere for 6 h. The completion of the reaction was monitored by TLC analysis (ethyl acetate/hexane = 1/1). The reaction flask was cooled to 0 °C and methanol was added, followed by stirring for additional 1 h. Then, EtOAc and saturated solution of NaHCO_3(aq)_ were added to the mixture. The crude product was extracted with EtOAc three times. The combined organic layers were washed with brine, dried over anhydrous MgSO_4_, filtered, and concentrated *in vacuo*. The residue was purified by silica gel flash column chromatography (ethyl acetate/hexane = 1/3) to afford pure compound **4α** (121 mg, 83%) as a cloudy white syrup. Single crystals (CCDC 2449041) suitable for X-ray analysis were obtained by recrystallization from dichloromethane/hexane, yielding exclusively the α-isomer **4α**. *R*_f_ = 0.42 (ethyl acetate/hexane = 1/1); mp 105–106 °C; [α]_D_^27^ – 55.4 (*c* = 1.0, CHCl_3_); IR (thin film): ν 2973, 1755, 1435, 1371, 1222, 1055 cm^–1^; ^1^H NMR (600 MHz, CDCl_3_) δ_H_ 5.43 (t, *J* = 9.8 Hz, 1H, H-4), 5.25 (d, *J* = 10.0 Hz, 1H, H-3), 5.09 (ddd, *J* = 6.1, 9.5, 10.8 Hz, 1H, H-5), 4.71 (d, *J* = 11.9 Hz, 1H, H-1a), 4.53 (d, *J* = 11.9 Hz, 1H, H-1b), 4.06 (dd, *J* = 6.1, 11.4 Hz, 1H, H-6a), 3.53 (t, *J* = 11.1 Hz, 1H, H-6b), 2.19 (s, 3H, Ac CH_3_), 2.07 (s, 3H, Ac CH_3_), 2.04 (s, 6H, Ac CH_3_), 2.01 (s, 3H, Ac CH_3_); ^13^C NMR (150 MHz, CDCl_3_) δ_C_ 170.1 (Ac C=O), 169.9 (Ac C=O), 169.7 (Ac C=O), 169.4 (Ac C=O), 167.8 (Ac C=O), 101.9 (C, C-2), 70.4 (CH, C-4), 68.9 (CH, C-3), 68.4 (CH, C-5), 62.2 (CH_2_, C-1), 61.1 (CH_2_, C-6), 21.5 (Ac CH_3_), 20.62 (2 × Ac CH_3_), 20.61 (Ac CH_3_), 20.5 (Ac CH_3_); HRMS–ESI (*m*/*z*): [M+NH_4_]^+^ calcd for C_16_H_22_O_11_NH_4_^+^ 408.1500; found, 408.1511.

### NMR Experiment

To monitor the progression of product formation, time-resolved ^1^H NMR spectroscopy was employed. Using **Method A**, following the addition of Cu­(OTf)_2_ to the reaction mixture, aliquots (∼100 μL) were withdrawn from the reaction flask at defined time intervals (e.g., 0.1, 0.5, 1, 5, 15, 30, 45, 60, 300 min). Each aliquot was immediately diluted with deuterated chloroform (CDCl_3_), and analyzed using ^1^H NMR spectroscopy. Spectra were recorded under consistent acquisition parameters to enable direct comparison of signal evolution over time. Product formation was assessed by tracking the emergence and integration of characteristic resonances corresponding to the desired product relative to starting material.

## Supplementary Material



## Data Availability

The data underlying this study are available in the published article and its Supporting Information.

## Notes

Experimental procedures, NMR, FT-IR, mass spectra, and X-ray crystallographic data for compounds **2** (CCDC 2449038), **3** (CCDC 2449039), **4α** (CCDC 2449041), and **9** (CCDC 2474135) (PDF)

## Notes

Deposition Numbers 2449038, 2449039, 2449041, and 2474135 contain the supplementary crystallographic data for this paper. These data can be obtained free of charge via the joint Cambridge Crystallographic Data Centre (CCDC) and Fachinformationszentrum Karlsruhe Access Structures service.
