# Supplementary material for: Copper(II) Trifluoromethanesulfonate as an Efficient, Versatile, and Dual-Purpose Catalyst for the Regioselective Acetylation of l‑Sorbose under Neat and Solvent-Directed Conditions
Source: ACS Omega. 2026 Jan 12;11(3):4712–21. doi: 10.1021/acsomega.5c11787 (PMC12854634; doi:10.1021/acsomega.5c11787)
Supplement: Supplementary file 1 [file ao5c11787_si_001.pdf]

*Supplementary Information*

# Copper(II) Trifluoromethanesulfonate as an Efficient, Versatile, and Dual-Purpose Catalyst for the Regioselective Acetylation of L-Sorbose under Neat and Solvent-Directed Conditions

Yu-An Chen,[‡,a,b] Jasper S. Dumalaog,[‡,a,c,d] Cheng-Hsiu Chang,[a] Fu-Chen Liu,*[b] and Shang-Cheng Hung*[a,c,d,e,f]

[a] Genomics Research Center, Academia Sinica, Taipei 115, Taiwan

[b] Department of Chemistry, National Dong Hwa University, Hualien 974, Taiwan

[c] Chemical Biology and Molecular Biophysics Program, Taiwan International Graduate Program (TIGP–CBMB), Academia Sinica, Taipei 115, Taiwan

[d] Department of Chemistry, National Tsing Hua University, Hsinchu 300, Taiwan

[e] Department of Chemistry, National Cheng Kung University, Tainan 701, Taiwan

[f] Department of Applied Science, National Taitung University, Taitung 950, Taiwan

* E-mail:

fcliu@mail.ndhu.edu.tw (Fu-Chen Liu)

schung@gate.sinica.edu.tw (Shang-Cheng Hung)

‡ These authors contributed equally to this work.

## Table of Contents


**Figure S1.** ORTEP drawing of **2** ........ S4

**Table S1.** Crystal data and structure refinement for **2**. ........ S4

**Figure S2.** ORTEP drawing of **3** ........ S6

**Table S2.** Crystal data and structure refinement for **3**. ........ S6

**Figure S3.** ORTEP drawing of **4*α*** ........ S8

**Table S3.** Crystal data and structure refinement for **4*α***. ........ S8

**Figure S4.** ORTEP drawing of **9** ........ S10

**Table S4.** Crystal data and structure refinement for **9**. ........ S10

**Figure S5.** $^{1}$H NMR spectrum of compound **2** ........ S12

**Figure S6.** $^{13}$C, DEPT-90, and DEPT-135 NMR spectrum of compound **2** ........ S13

**Figure S7.** $^{1}$H–$^{1}$H COSY NMR spectrum of compound **2** ........ S14

**Figure S8.** $^{13}$C–$^{1}$H HSQC NMR spectrum of compound **2** ........ S15

**Figure S9.** FT–IR spectrum of compound **2** ........ S16

**Figure S10.** HRMS–ESI spectrum of compound **2** ........ S17

**Figure S11.** $^{1}$H NMR spectrum of compound **3** ........ S18

**Figure S12.** $^{13}$C, DEPT-90, and DEPT-135 NMR spectrum of compound **3** ........ S19

**Figure S13.** $^{1}$H–$^{1}$H COSY NMR spectrum of compound **3** ........ S20

**Figure S14.** $^{13}$C–$^{1}$H HSQC NMR spectrum of compound **3** ........ S21

**Figure S15.** $^{13}$C–$^{1}$H HMBC NMR spectrum of compound **3** ........ S22

**Figure S16.** $^{1}$H–$^{1}$H NOESY NMR spectrum of compound **3** ........ S23

**Figure S17.** FT–IR spectrum of compound **3** ........ S24

**Figure S18.** HRMS–ESI spectrum of compound **3** ........ S25

**Figure S19.** $^{1}$H NMR spectrum of compound **4*α*+4*β*** ........ S26

**Figure S20.** $^{13}$C, DEPT-90, and DEPT-135 NMR spectrum of compound **4*α*+4*β*** ........ S27

**Figure S21.** $^{1}$H–$^{1}$H COSY NMR spectrum of compound **4*α*+4*β*** ........ S28

**Figure S22.** $^{13}$C–$^{1}$H HSQC NMR spectrum of compound **4*α*+4*β*** ........ S29

**Figure S23.** 1D TOCSY NMR spectrum of compound **4*α*+4*β*** ........ S30

**Figure S24.** FT–IR spectrum of compound **4*α*+4*β*** ........ S31

**Figure S25.** HRMS–ESI spectrum of compound **4*α*+4*β*** ........ S32

**Figure S26.** $^{1}$H NMR spectrum of compound **4*α*** ........ S33

**Figure S27.** $^{13}$C, DEPT-90, and DEPT-135 NMR spectrum of compound **4*α*** ........ S34

**Figure S28.** $^{1}$H–$^{1}$H COSY NMR spectrum of compound **4*α*** ........ S35

**Figure S29.** $^{13}$C–$^{1}$H HSQC NMR spectrum of compound **4*α*** ........ S36

**Figure S30.** $^1H$–$^1H$ NOESY NMR spectrum of compound **4*α*** ............................................ S37
**Figure S31.** FT–IR spectrum of compound **4*α*** ..................................................................... S38
**Figure S32.** HRMS–ESI spectrum of compound **4*α***............................................................ S39
**Figure S33.** $^1H$ NMR spectrum of compound **9** .................................................................... S40
**Figure S34.** $^{13}C$ NMR spectrum of compound **9** .................................................................. S41

**X-ray Analysis**

The single X-ray quality crystals of **2** were grown from ethyl acetate by slow evaporation.

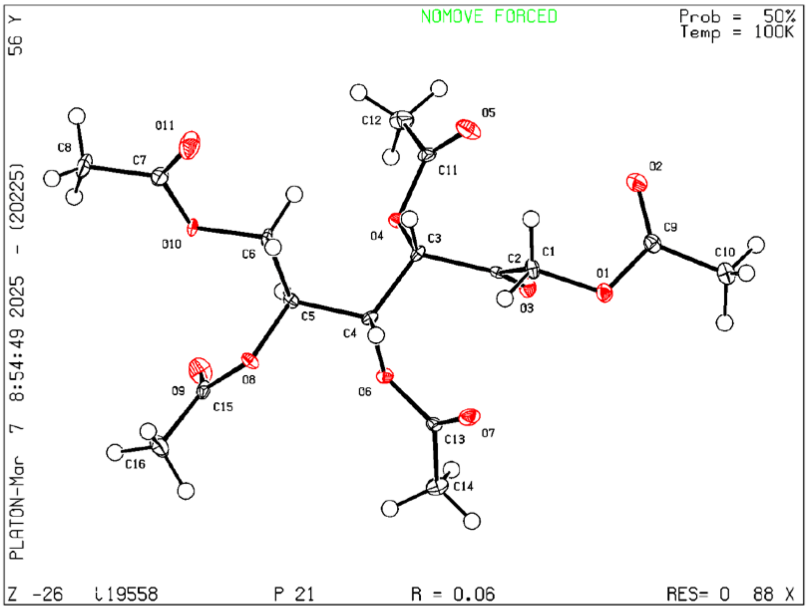


**Figure S1.** ORTEP drawing of **2** (CCDC 2449038, 50% thermal probability).

**Table S1.** Crystal data and structure refinement for **2**.

| | | |
|---|---|---|
| Empirical formula | $C_{16}H_{22}O_{11}$ | |
| Formula weight | 390.33 | |
| Temperature | 100.0(2) K | |
| Wavelength | 1.54178 Å | |
| Crystal system | Monoclinic | |
| Space group | P 21 | |
| Unit cell dimensions | a = 6.09510(10) Å | α = 90°. |
| | b = 20.2089(5) Å | β = 105.4500(10)°. |
| | c = 7.8102(2) Å | γ = 90°. |
| Volume | 927.26(4) Å$^3$ | |
| Z | 2 | |

| | |
|---|---|
| Density (calculated) | 1.398 $Mg/m^3$ |
| Absorption coefficient | 1.035 $mm^{-1}$ |
| F(000) | 412 |
| Crystal size | 0.535 x 0.474 x 0.334 $mm^3$ |
| Theta range for data collection | 5.878 to 68.171°. |
| Index ranges | -7<=h<=7, -24<=k<=24, -9<=l<=9 |
| Reflections collected | 19417 |
| Independent reflections | 3237 [R(int) = 0.0393] |
| Completeness to theta = 67.679∞ | 98.8 % |
| Absorption correction | Numerical |
| Max. and min. transmission | 1 and 0.6944 |
| Refinement method | Full-matrix least-squares on $F^2$ |
| Data / restraints / parameters | 3237 / 181 / 249 |
| Goodness-of-fit on $F^2$ | 1.167 |
| Final R indices [I>2sigma(I)] | R1 = 0.0645, wR2 = 0.1528 |
| R indices (all data) | R1 = 0.0645, wR2 = 0.1528 |
| Absolute structure parameter | -0.18(13) |
| Extinction coefficient | n/a |
| Largest diff. peak and hole | 0.486 and -0.602 e. $Å^{-3}$ |

The single X-ray quality crystals of **3** were grown from dichloromethane/hexane by slow evaporation.

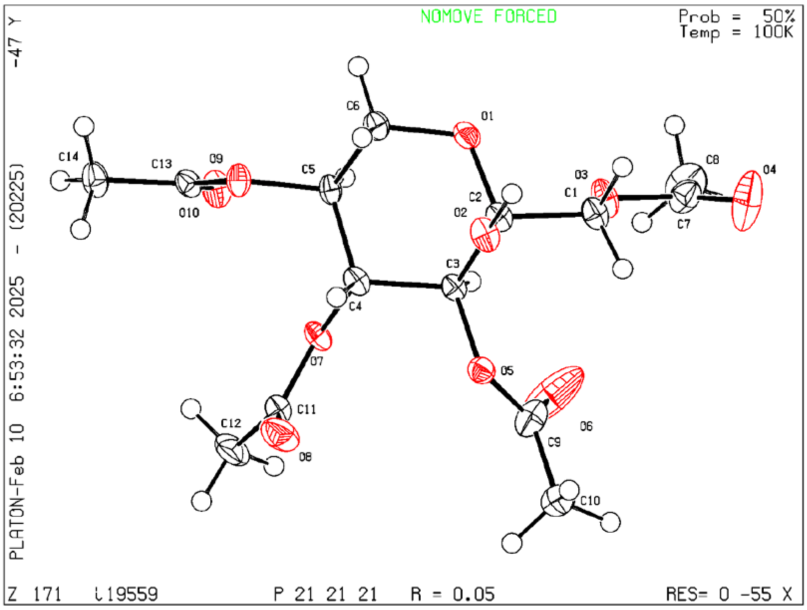

**Figure S2.** ORTEP drawing of **3** (CCDC 2449039, 50% thermal probability).

**Table S2.** Crystal data and structure refinement for **3**.

| | | |
|---|---|---|
| Empirical formula | $C_{14}H_{20}O_{10}$ | |
| Formula weight | 348.30 | |
| Temperature | 100.0(2) K | |
| Wavelength | 1.54178 Å | |
| Crystal system | Orthorhombic | |
| Space group | P 21 21 21 | |
| Unit cell dimensions | a = 8.4162(2) Å | α = 90°. |
| | b = 9.4977(2) Å | β = 90°. |
| | c = 20.9970(4) Å | γ = 90°. |
| Volume | 1678.39(6) $Å^3$ | |
| Z | 4 | |

| | |
|---|---|
| Density (calculated) | 1.378 $Mg/m^3$ |
| Absorption coefficient | 1.028 $mm^{-1}$ |
| F(000) | 736 |
| Crystal size | 0.257 x 0.208 x 0.196 $mm^3$ |
| Theta range for data collection | 5.111 to 68.292°. |
| Index ranges | -9<=h<=10, -11<=k<=11, -25<=l<=25 |
| Reflections collected | 19976 |
| Independent reflections | 3051 [R(int) = 0.0360] |
| Completeness to theta = 67.679∞ | 99.8 % |
| Absorption correction | Numerical |
| Max. and min. transmission | 1 and 0.6227 |
| Refinement method | Full-matrix least-squares on $F^2$ |
| Data / restraints / parameters | 3051 / 174 / 224 |
| Goodness-of-fit on $F^2$ | 1.172 |
| Final R indices [I>2sigma(I)] | R1 = 0.0462, wR2 = 0.1169 |
| R indices (all data) | R1 = 0.0464, wR2 = 0.1174 |
| Absolute structure parameter | -0.04(10) |
| Extinction coefficient | 0.0132(16) |
| Largest diff. peak and hole | 0.408 and -0.452 e. $Å^{-3}$ |

The single X-ray quality crystals of **4*α*** were grown from dichloromethane/hexane by slow evaporation.

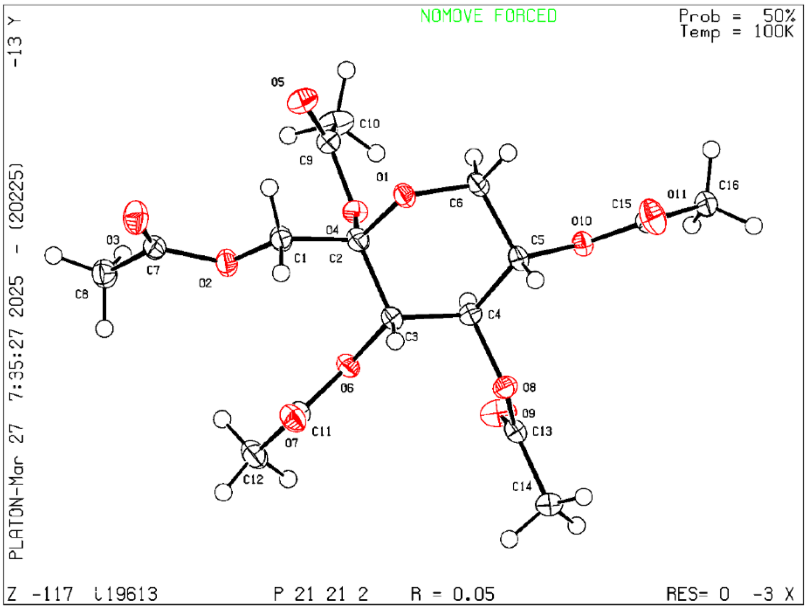


**Figure S3.** ORTEP drawing of **4*α*** (CCDC 2449041, 50% thermal probability).

**Table S3.** Crystal data and structure refinement for **4*α***.

| | | |
|---|---|---|
| Empirical formula | $C_{16}H_{22}O_{11}$ | |
| Formula weight | 390.33 | |
| Temperature | 100.0(2) K | |
| Wavelength | 0.71073 Å | |
| Crystal system | Orthorhombic | |
| Space group | P 21 21 2 | |
| Unit cell dimensions | a = 14.3498(3) Å | α = 90°. |
| | b = 22.8762(6) Å | β = 90°. |
| | c = 5.62210(10) Å | γ = 90°. |
| Volume | 1845.56(7) $Å^3$ | |

| | |
|---|---|
| Z | 4 |
| Density (calculated) | 1.405 Mg/m$^3$ |
| Absorption coefficient | 0.120 mm$^{-1}$ |
| F(000) | 824 |
| Crystal size | 0.506 x 0.467 x 0.326 mm$^3$ |
| Theta range for data collection | 1.675 to 25.724°. |
| Index ranges | -17<=h<=17, -27<=k<=27, -6<=l<=6 |
| Reflections collected | 20337 |
| Independent reflections | 3502 [R(int) = 0.0381] |
| Completeness to theta = 25.242∞ | 99.6 % |
| Absorption correction | Numerical |
| Max. and min. transmission | 1 and 0.869 |
| Refinement method | Full-matrix least-squares on F$^2$ |
| Data / restraints / parameters | 3502 / 195 / 250 |
| Goodness-of-fit on F$^2$ | 1.196 |
| Final R indices [I>2sigma(I)] | R1 = 0.0460, wR2 = 0.1160 |
| R indices (all data) | R1 = 0.0464, wR2 = 0.1163 |
| Absolute structure parameter | 0.1(6) |
| Extinction coefficient | 0.074(6) |
| Largest diff. peak and hole | 0.386 and -0.474 e. Å$^{-3}$ |

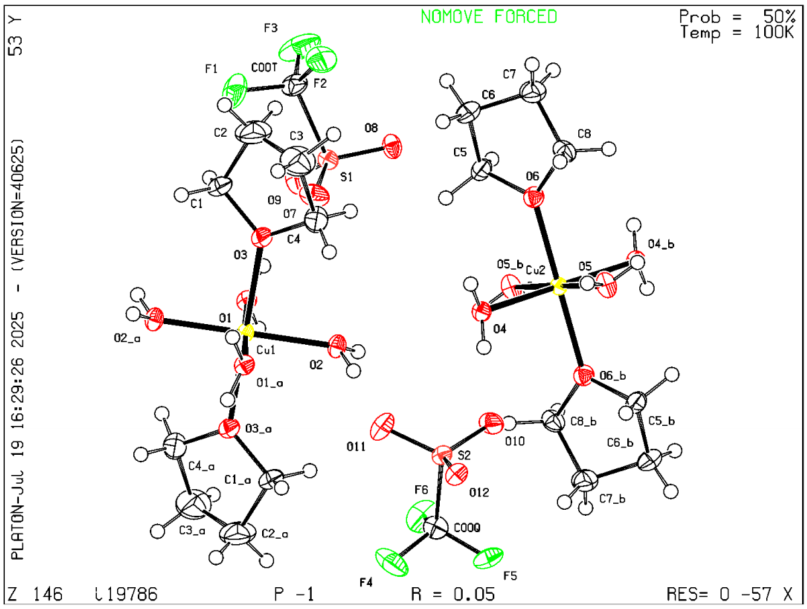


**Figure S4.** ORTEP drawing of **9** (CCDC 2474135, 50% thermal probability).

**Table S4.** Crystal data and structure refinement for **9**.

| | | |
|---|---|---|
| Empirical formula | $C_{10}H_{24}CuF_6O_{12}S_2$ | |
| Formula weight | 577.95 | |
| Temperature | 100.0(2) K | |
| Wavelength | 0.71073 Å | |
| Crystal system | Triclinic | |
| Space group | P -1 | |
| Unit cell dimensions | a = 8.7689(14) Å | α = 74.502(4)°. |
| | b = 10.8778(17) Å | β = 81.118(4)°. |
| | c = 12.2006(19) Å | γ = 88.867(4)°. |
| Volume | 1107.7(3) Å$^3$ | |
| Z | 2 | |
| Density (calculated) | 1.733 Mg/m$^3$ | |
| Absorption coefficient | 1.277 mm$^{-1}$ | |

| | |
|---|---|
| F(000) | 590 |
| Crystal size | 0.579 x 0.541 x 0.473 $mm^3$ |
| Theta range for data collection | 1.943 to 24.996°. |
| Index ranges | -10<=h<=10, -12<=k<=12, -14<=l<=14 |
| Reflections collected | 23446 |
| Independent reflections | 3909 [R(int) = 0.0737] |
| Completeness to theta = 24.996° | 99.8 % |
| Absorption correction | Numerical |
| Max. and min. transmission | 1 and 0.7993 |
| Refinement method | Full-matrix least-squares on $F^2$ |
| Data / restraints / parameters | 3909 / 212 / 307 |
| Goodness-of-fit on $F^2$ | 1.060 |
| Final R indices [I>2sigma(I)] | R1 = 0.0550, wR2 = 0.1380 |
| R indices (all data) | R1 = 0.0599, wR2 = 0.1454 |
| Extinction coefficient | n/a |
| Largest diff. peak and hole | 1.821 and -1.066 e.$Å^{-3}$ |

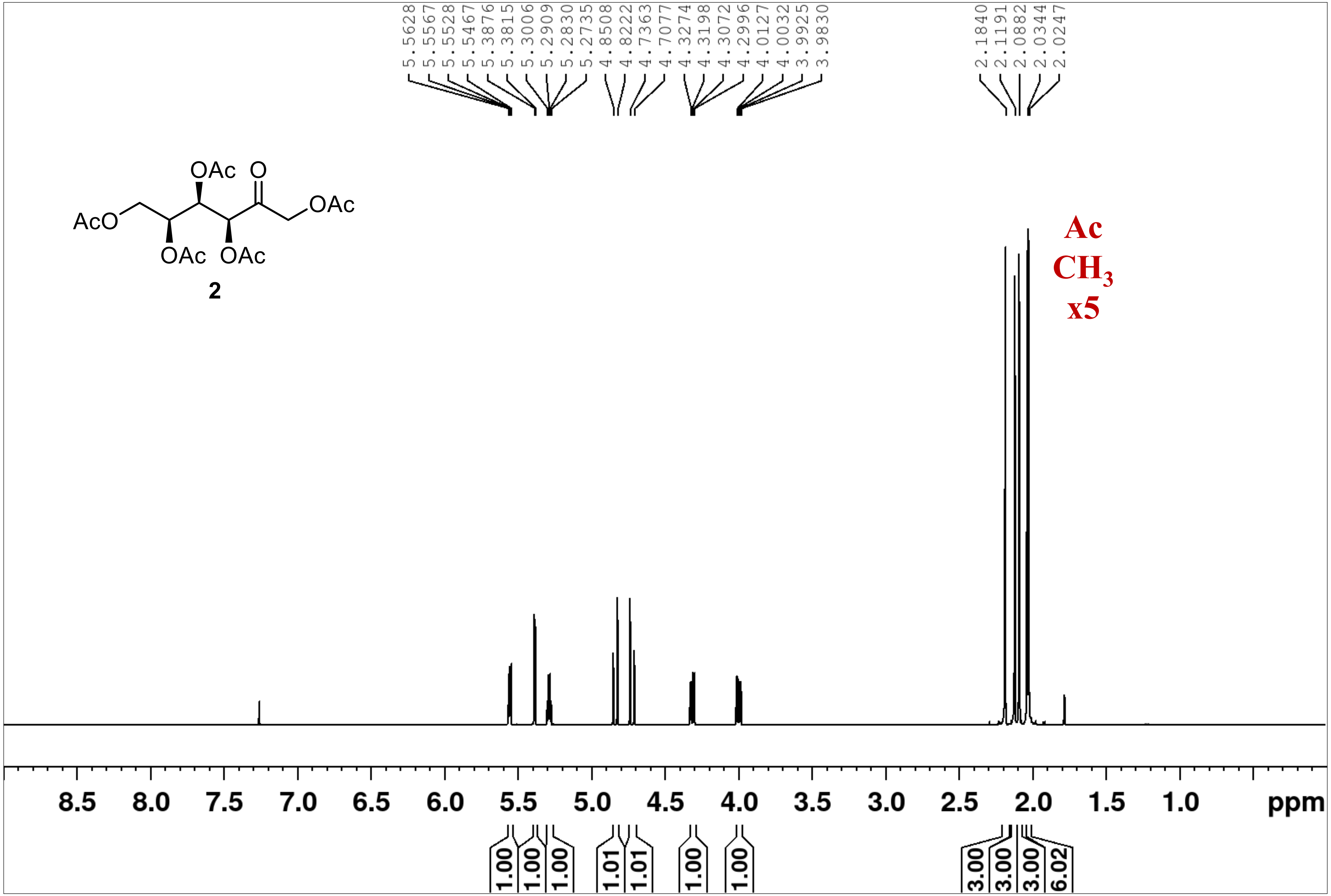


**Figure S5.** $^{1}$H NMR spectrum of compound **2**.

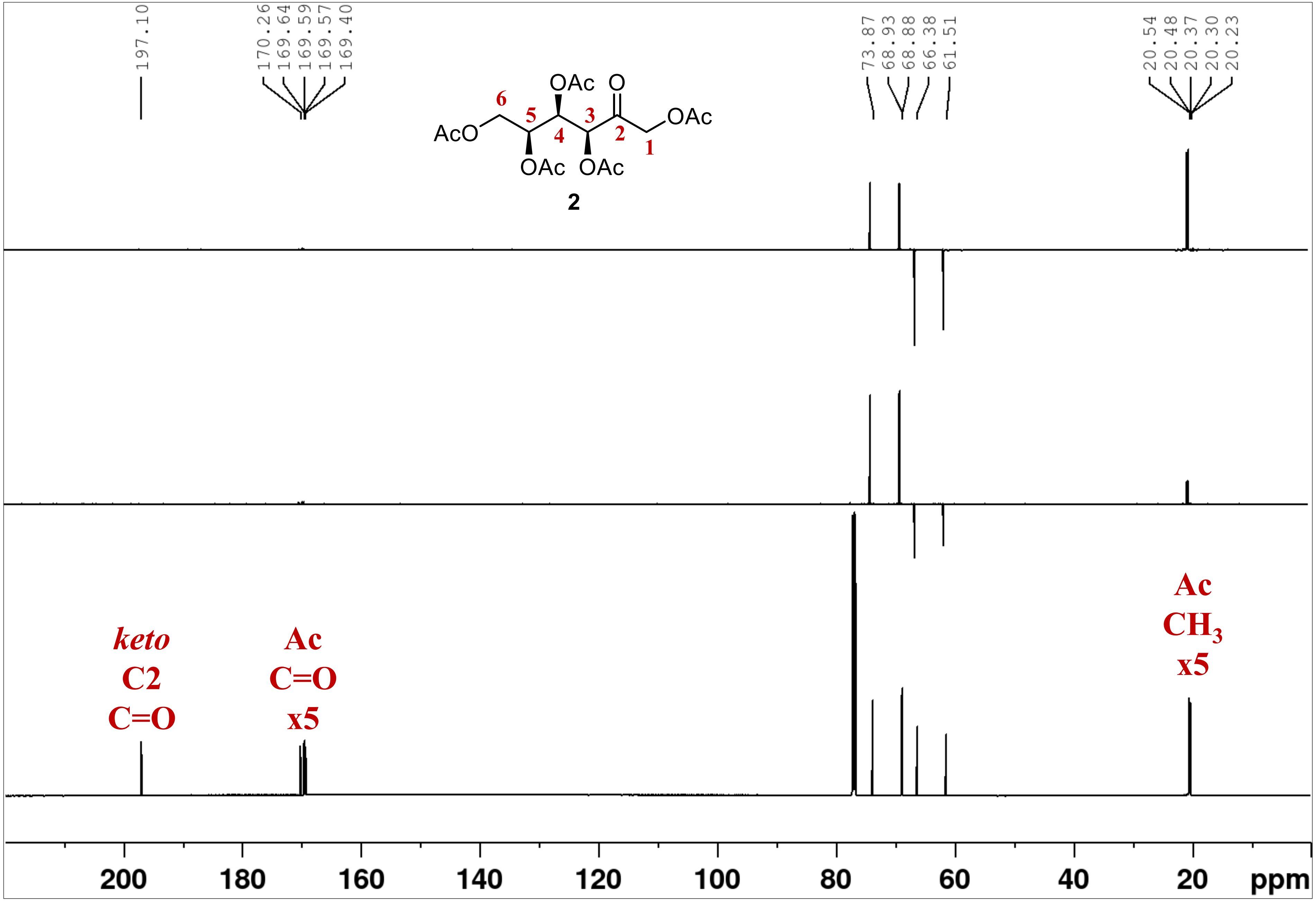


**Figure S6.** $^{13}C$, DEPT-90, and DEPT-135 NMR spectrum of compound **2**.

# $^1$H-$^1$H COSY

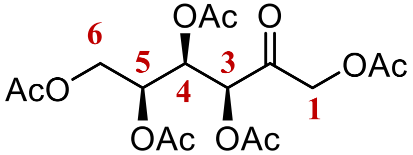


**2**

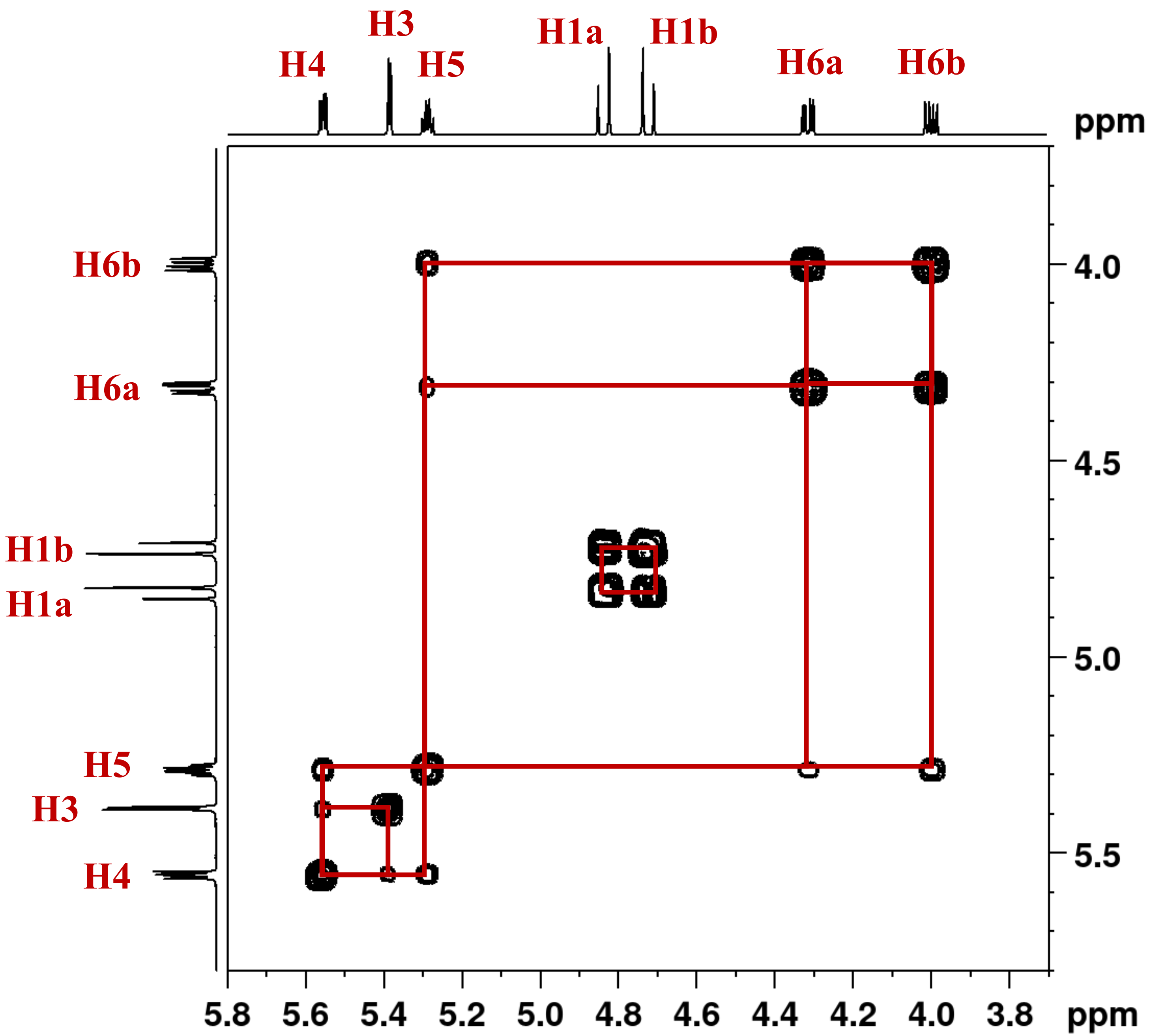


**Figure S7.** $^1$H–$^1$H COSY NMR spectrum of compound **2**.

# $^{13}$C-$^{1}$H HSQC

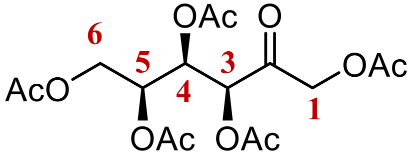

**2**

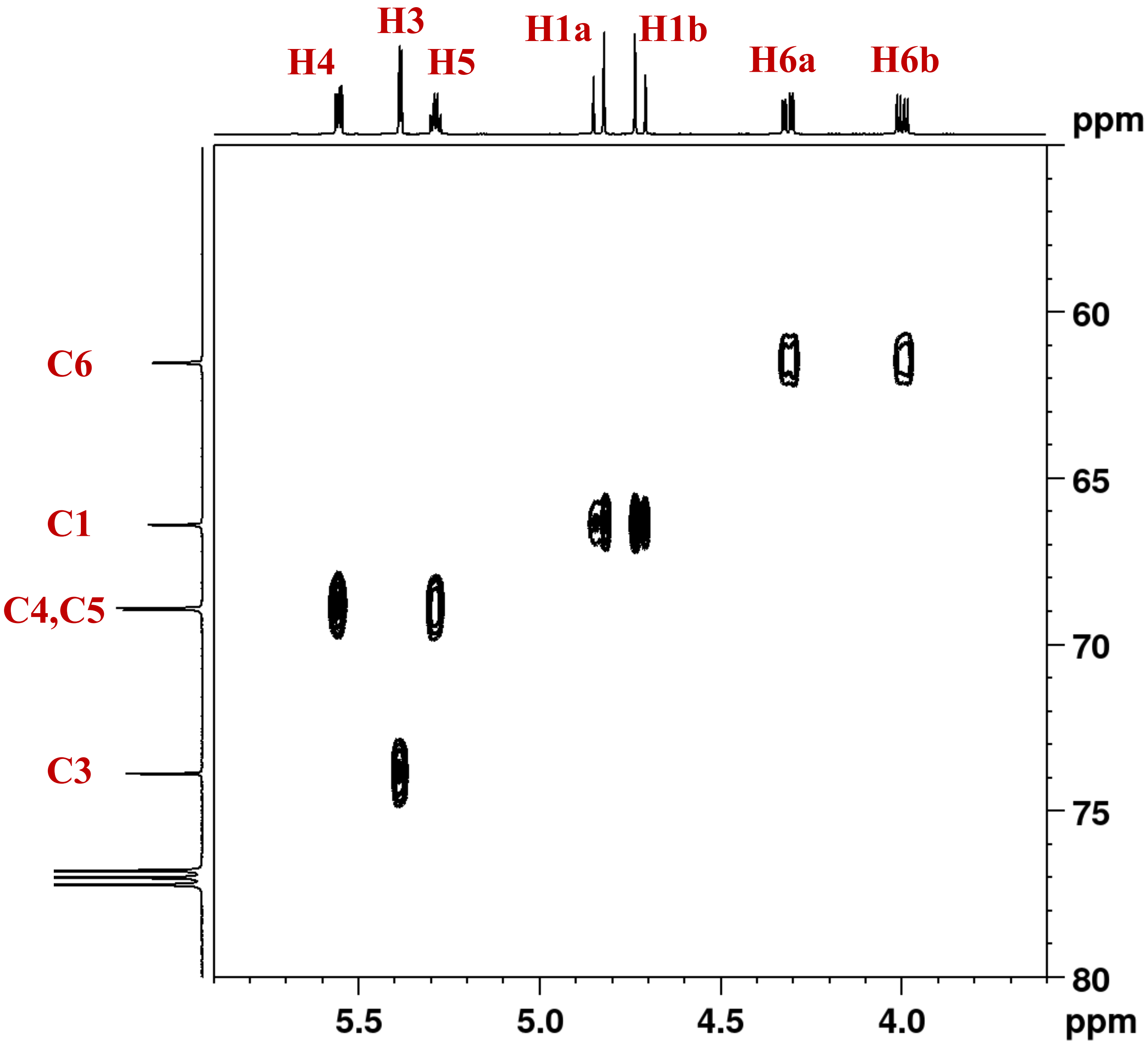


**Figure S8.** $^{13}$C–$^{1}$H HSQC NMR spectrum of compound **2**.

# IR Spectrum

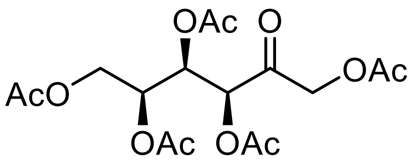


**2**

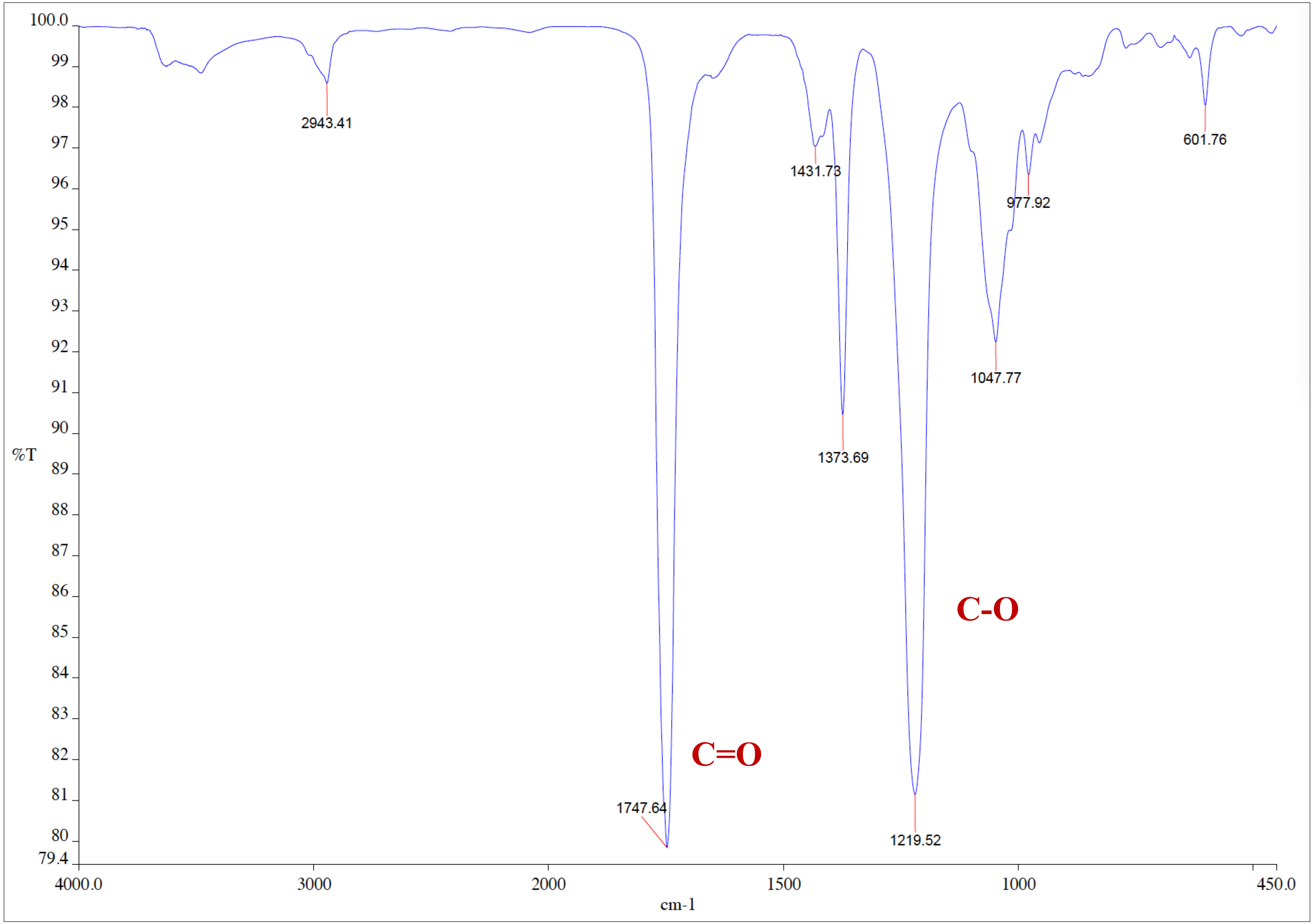


**Figure S9.** FT-IR spectrum of compound **2**.

# HRMS–ESI

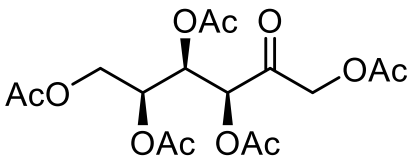


**2**

**Calculated : 408.1500**

**Found : 408.1505**

**Mass Error : 1.23 ppm**

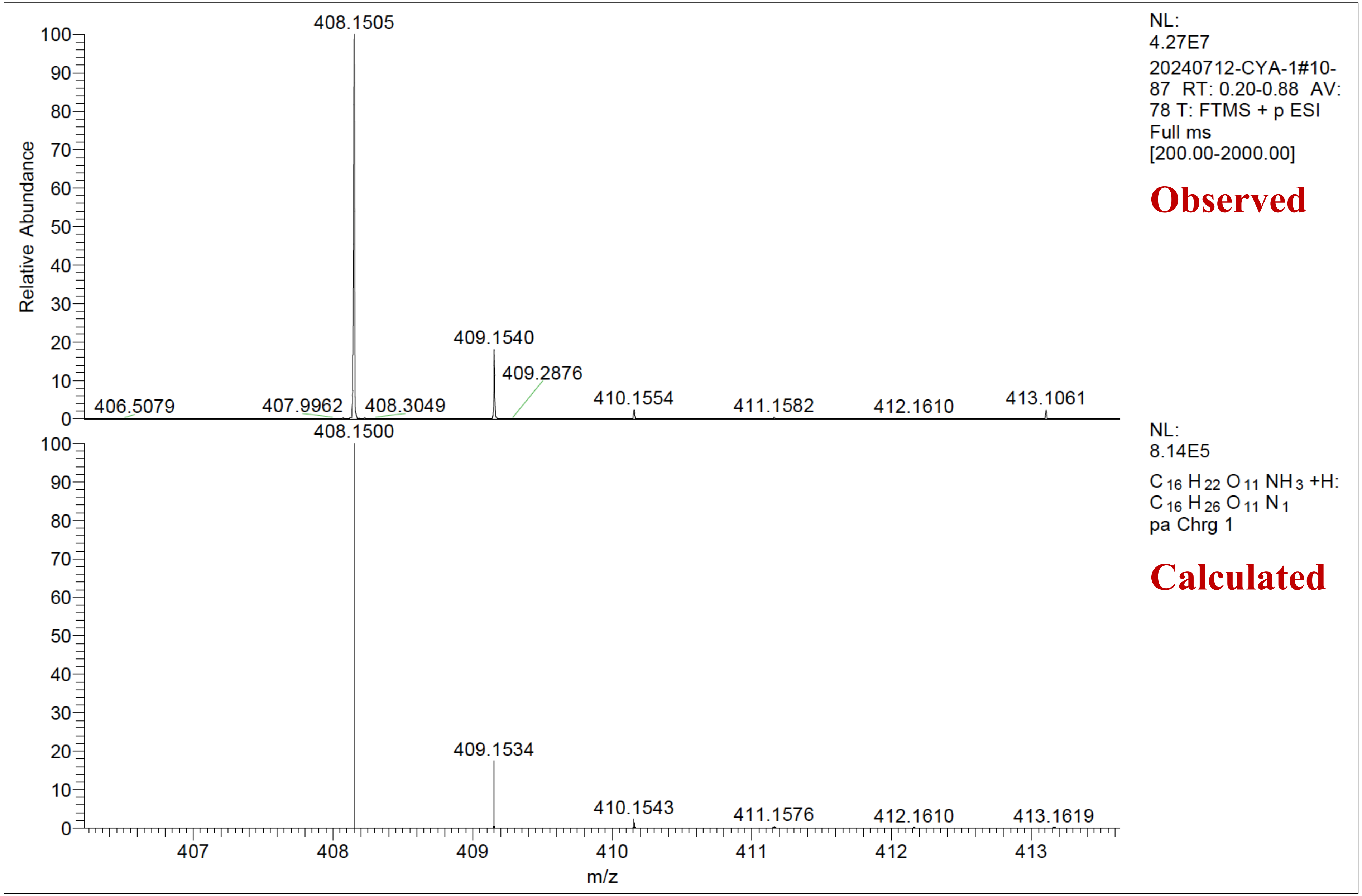


**Figure S10.** HRMS–ESI spectrum of compound **2**.

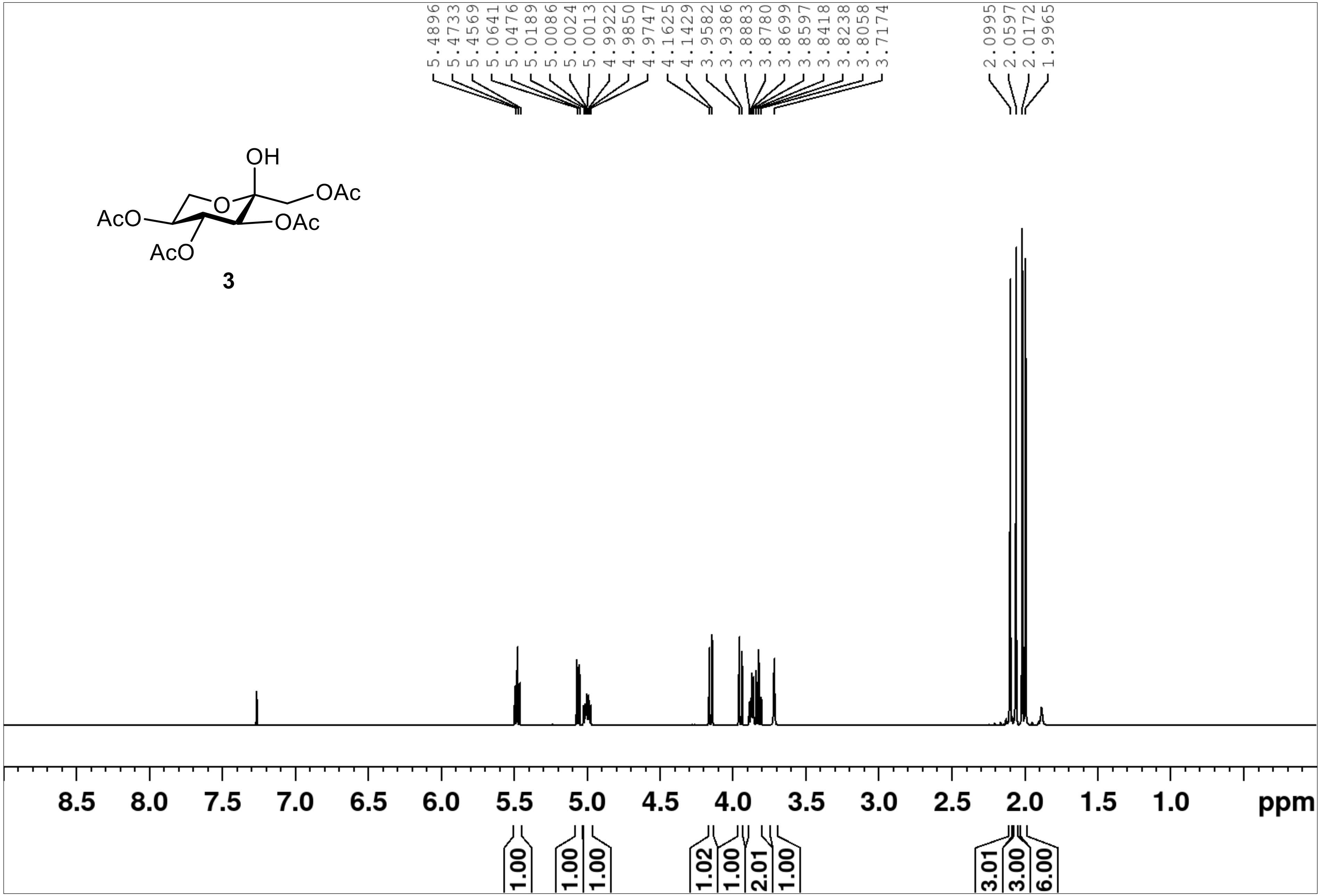


**Figure S11.** $^{1}$H NMR spectrum of compound **3**.

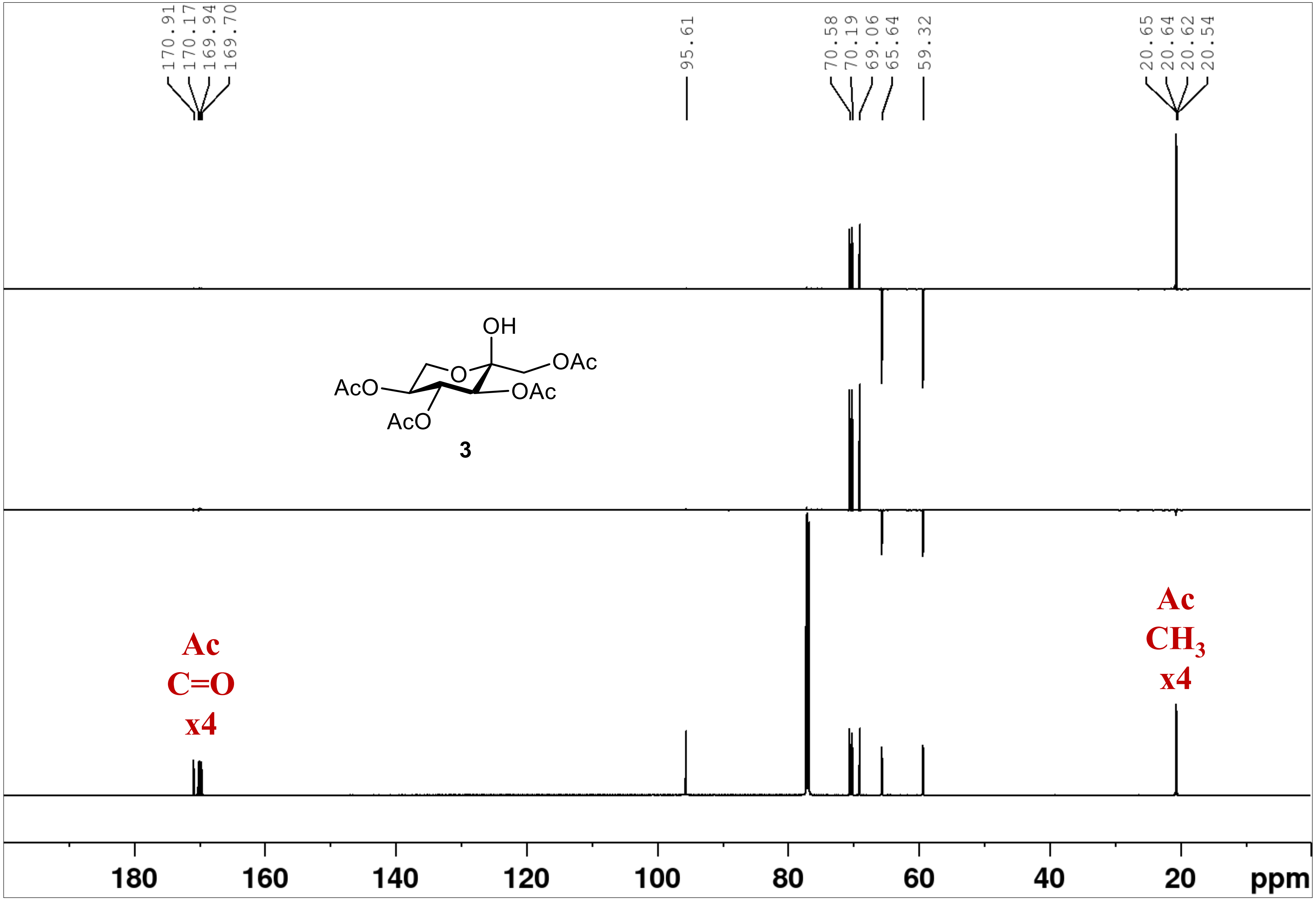


**Figure S12.** $^{13}$C, DEPT-90, DEPT-135 NMR spectrum of compound **3**.

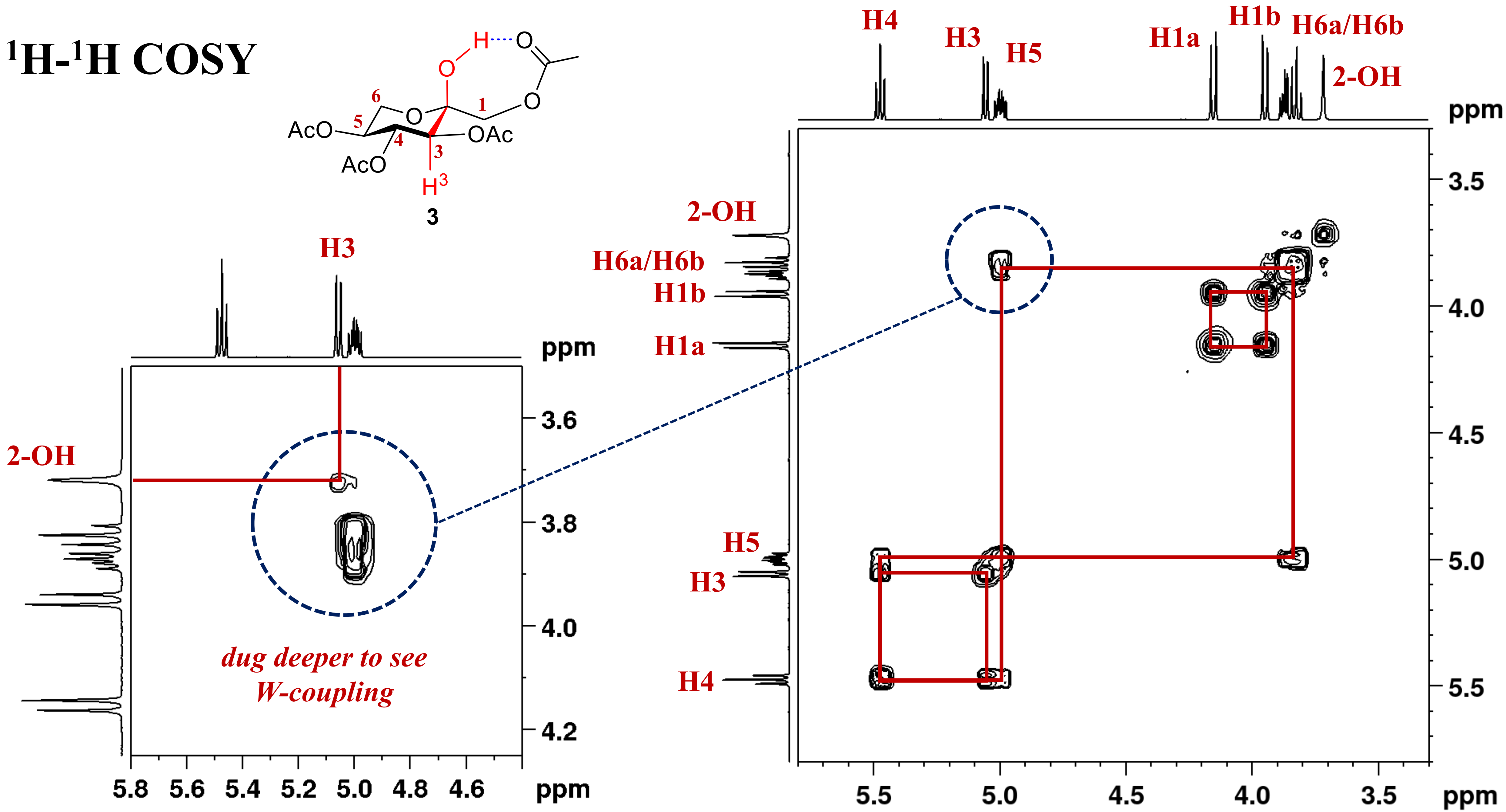


**Figure S13.** $^1$H–$^1$H COSY NMR spectrum of compound **3**.

# $^{13}C$-$^{1}H$ HSQC

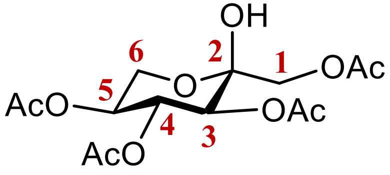


**3**

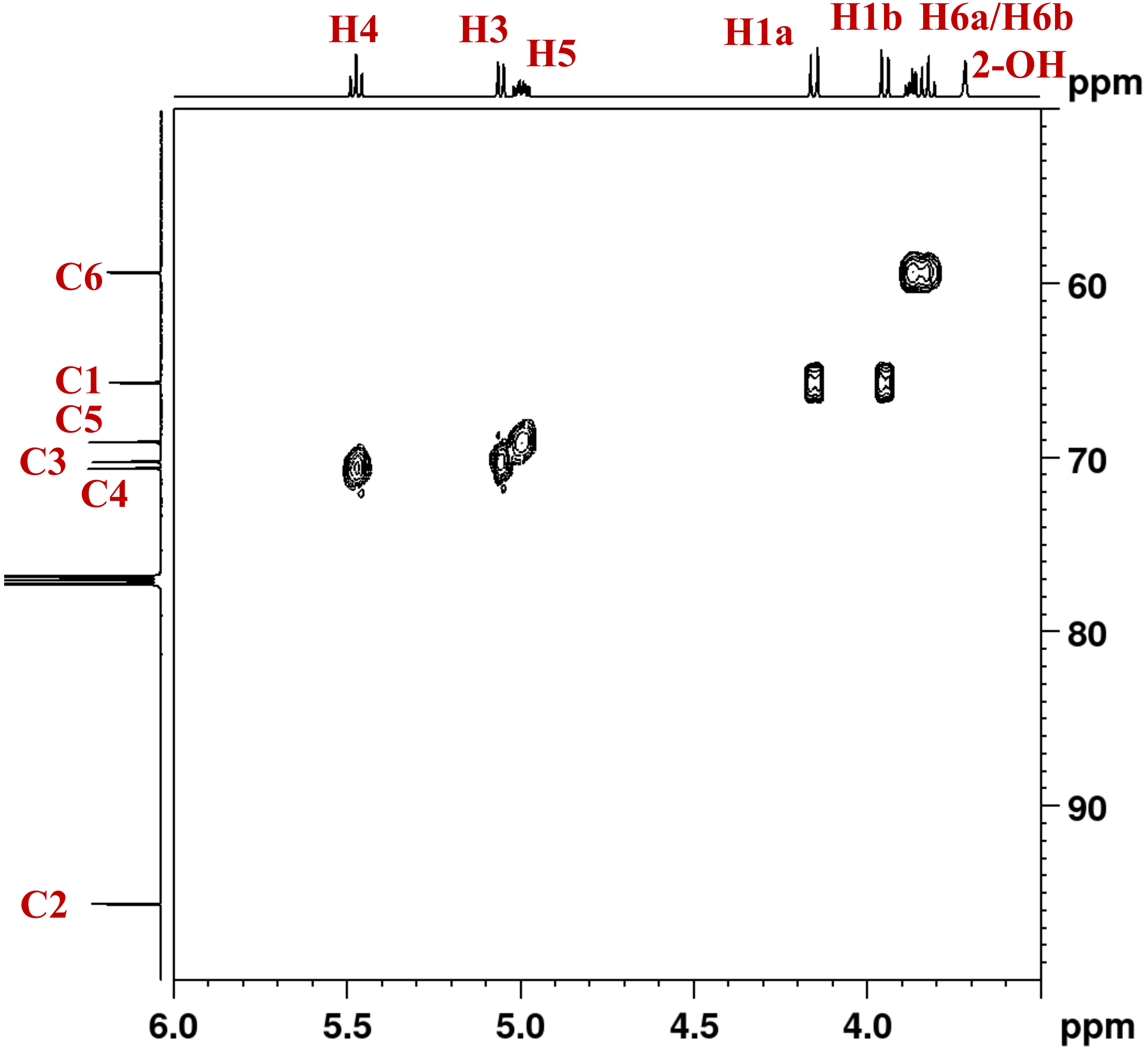


**Figure S14.** $^{13}C$–$^{1}H$ HSQC NMR spectrum of compound **3**.

# $^{13}$C-$^{1}$H HMBC

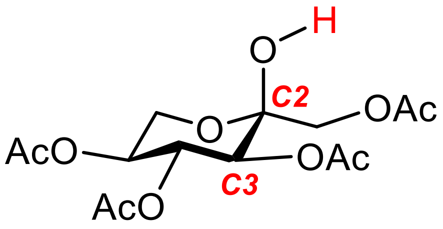


**3**

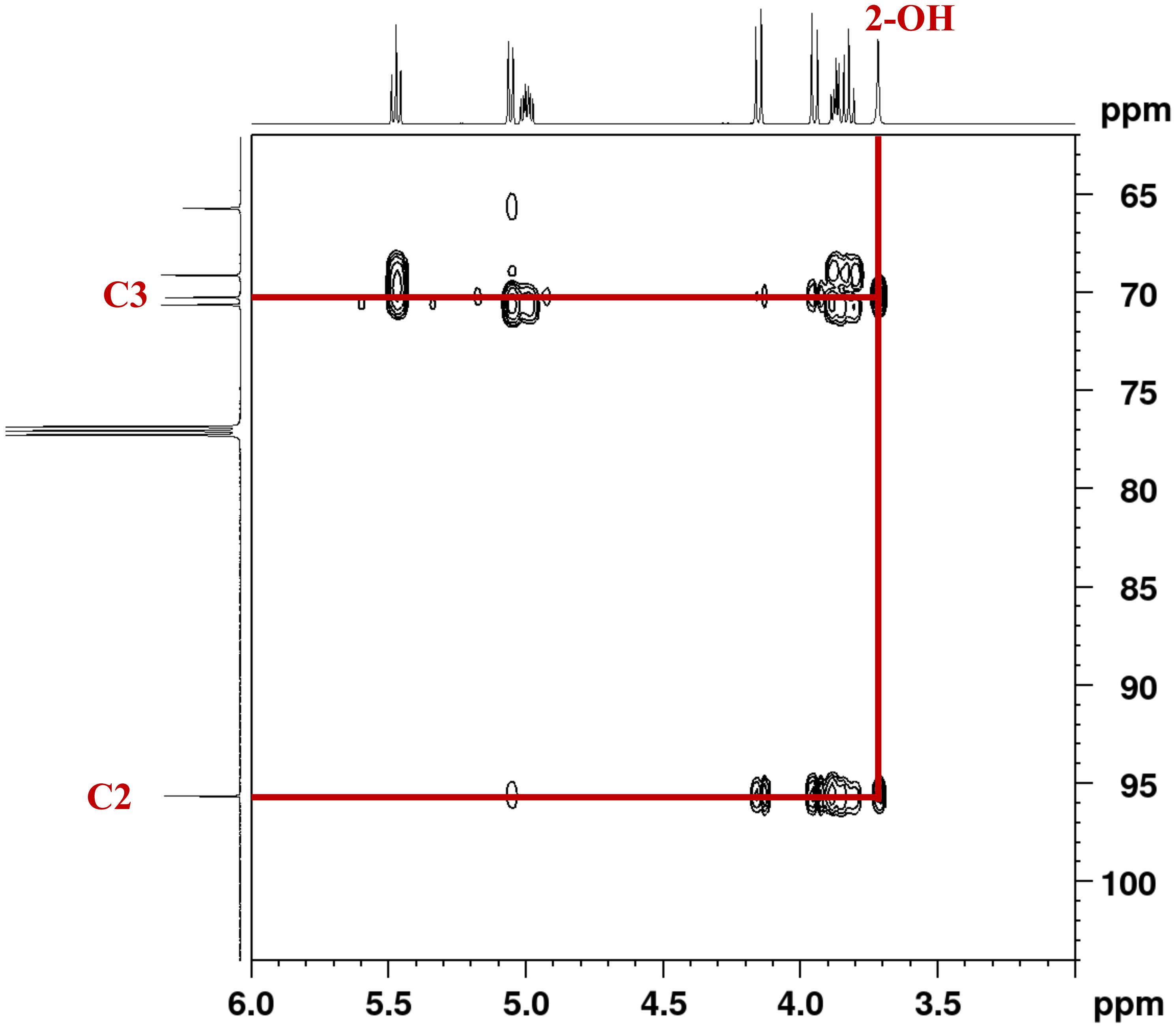


**Figure S15.** $^{13}$C–$^{1}$H HMBC NMR spectrum of compound **3**.

# $^{1}H$-$^{1}H$ NOESY

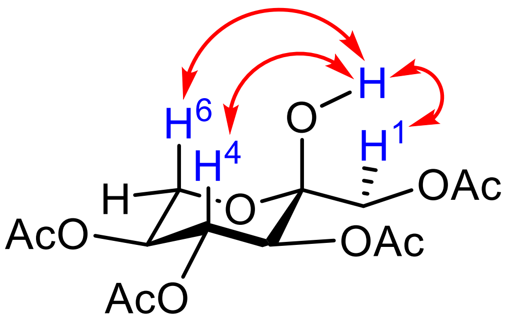


**3**

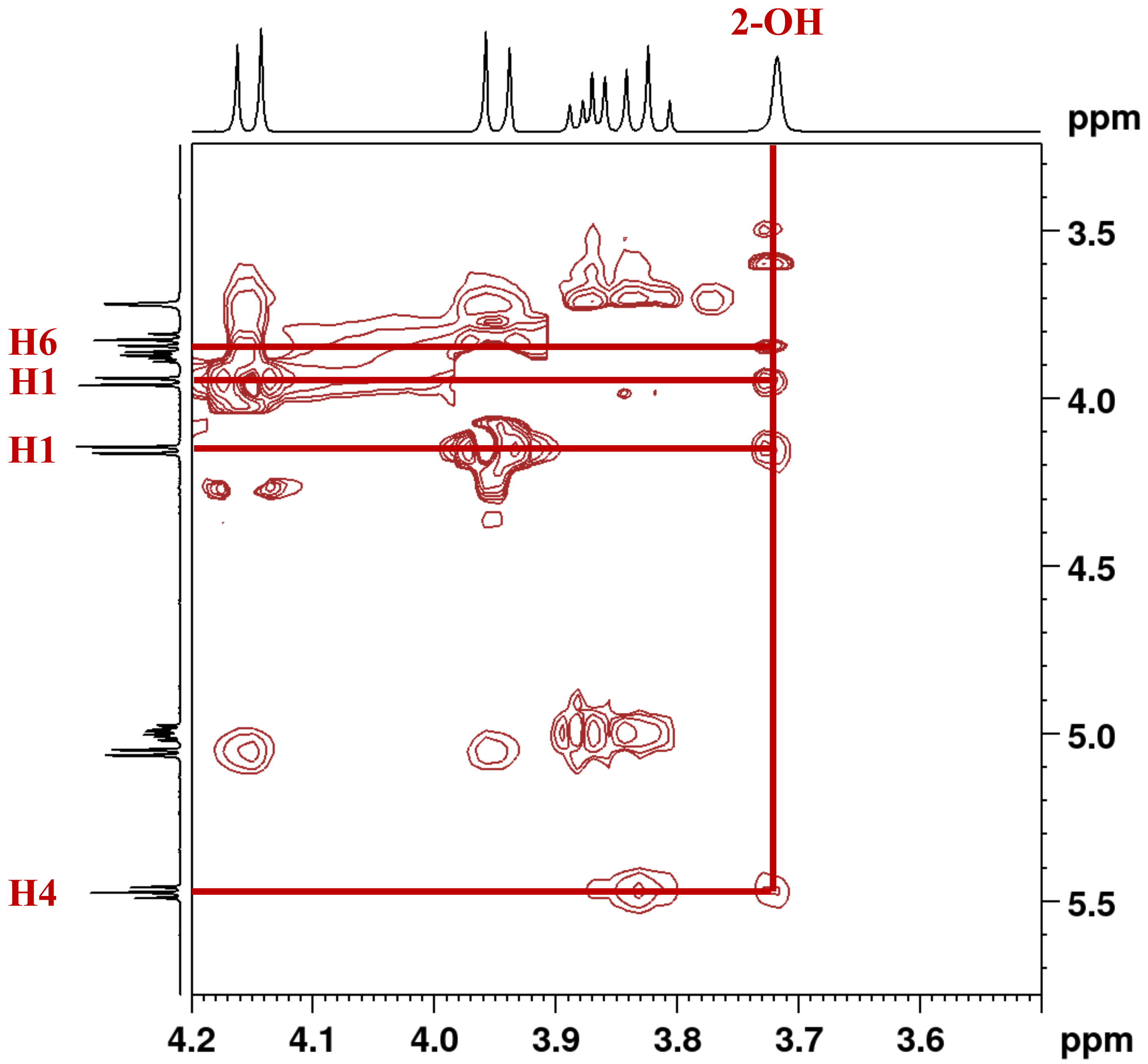


**Figure S16.** $^{1}H$–$^{1}H$ NOESY NMR spectrum of compound **3**.

# IR Spectrum

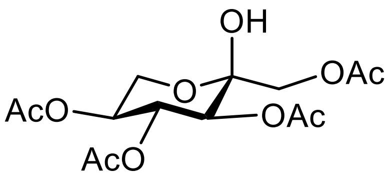


**3**

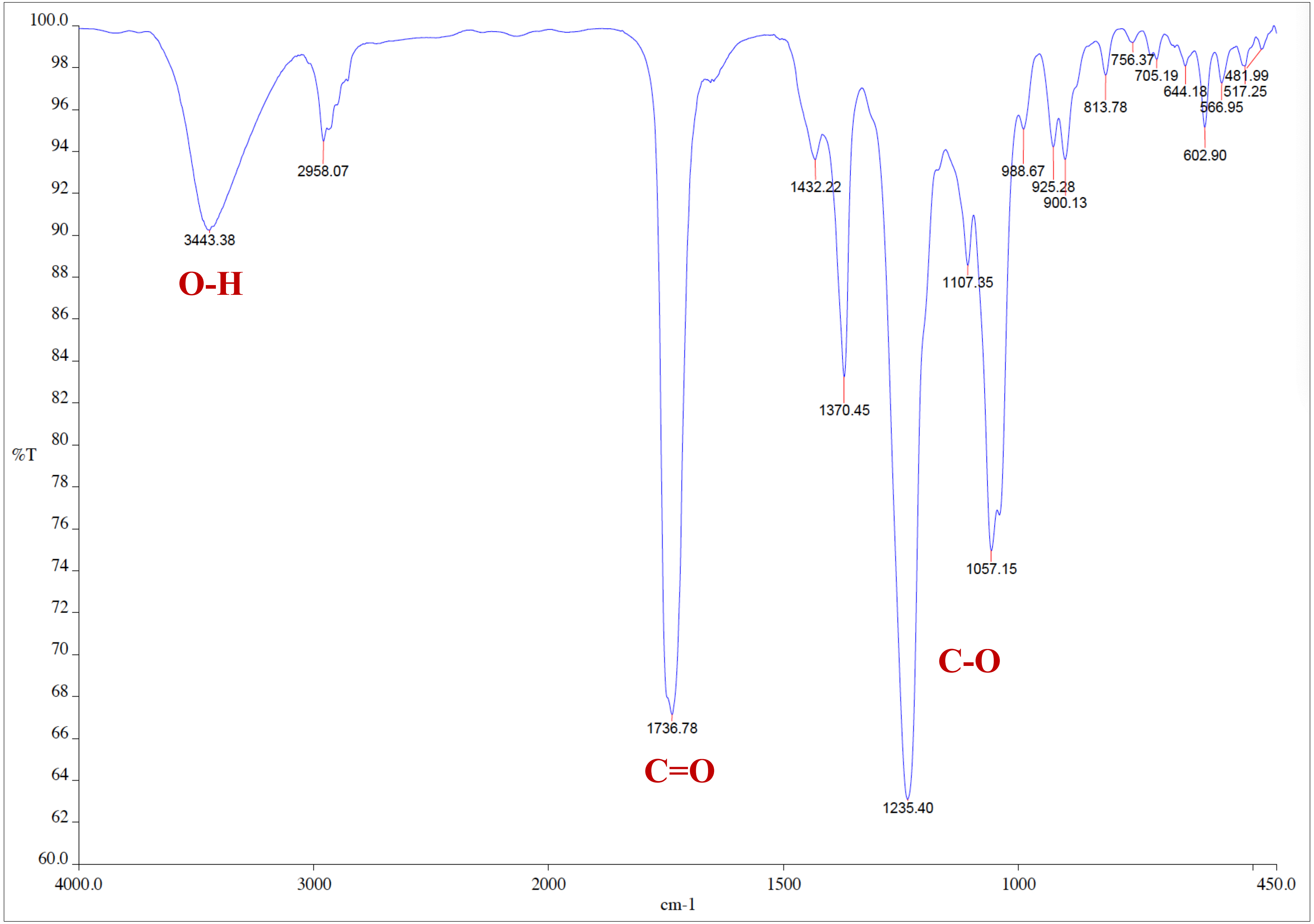


**Figure S17.** FT–IR spectrum of compound **3**.

# HRMS–ESI

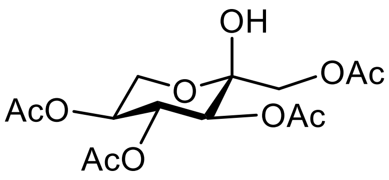


**3**

**Calculated : 366.1395**

**Found : 366.1401**

**Mass Error : 1.64 ppm**

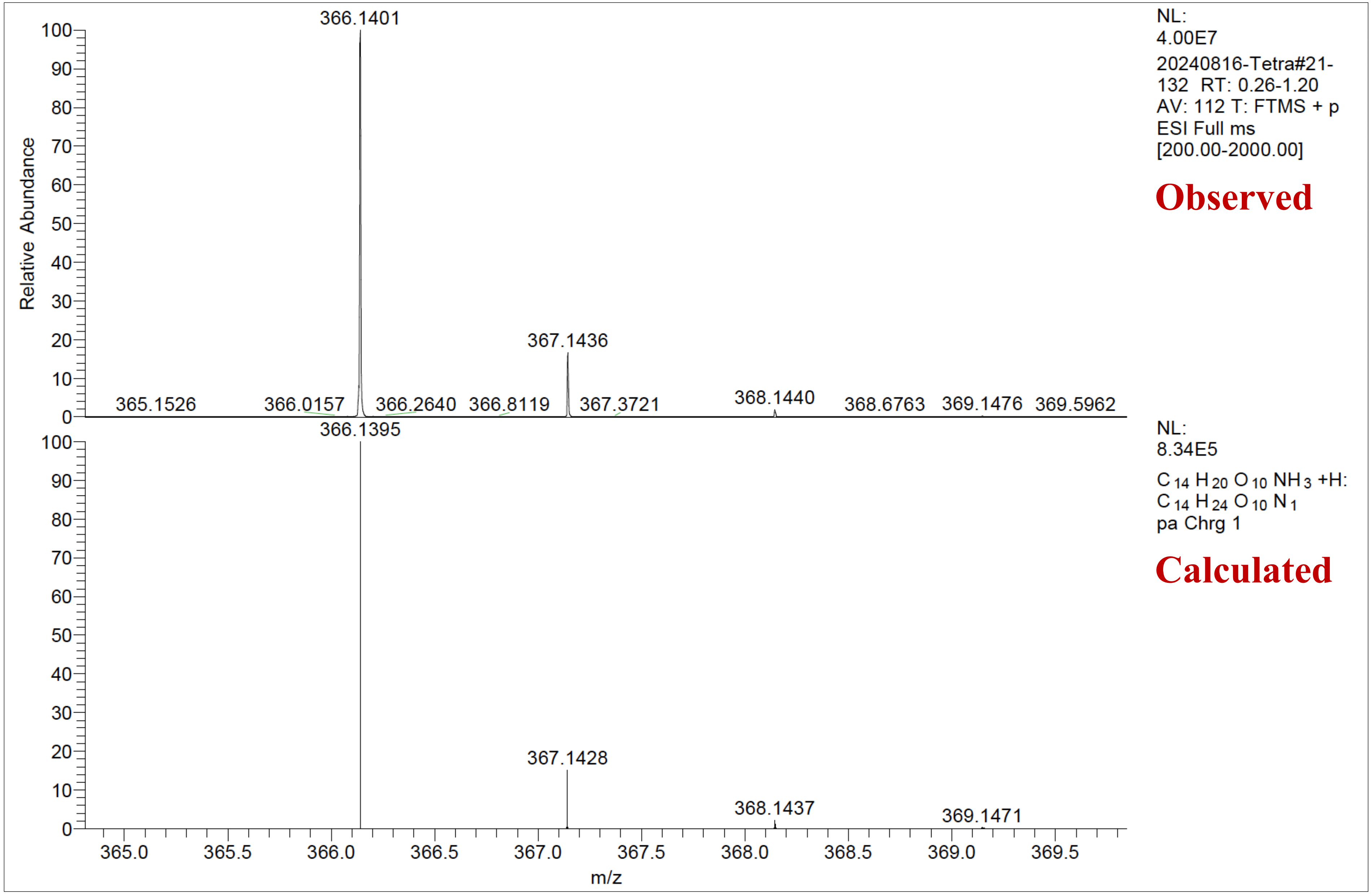


**Figure S18.** HRMS–ESI spectrum of compound **3**.

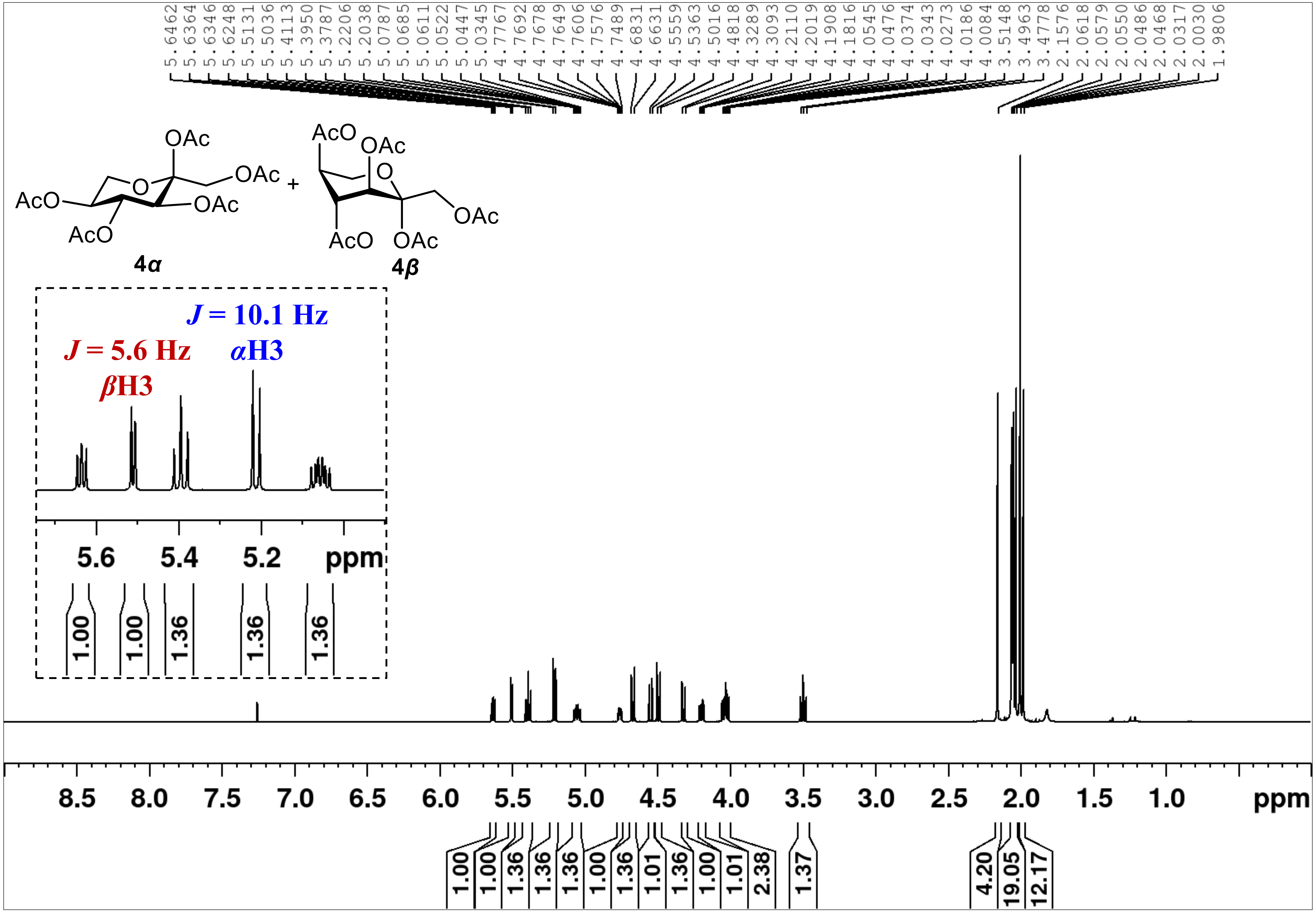


**Figure S19.** $^{1}$H NMR spectrum of compound **4*α*+4*β***.

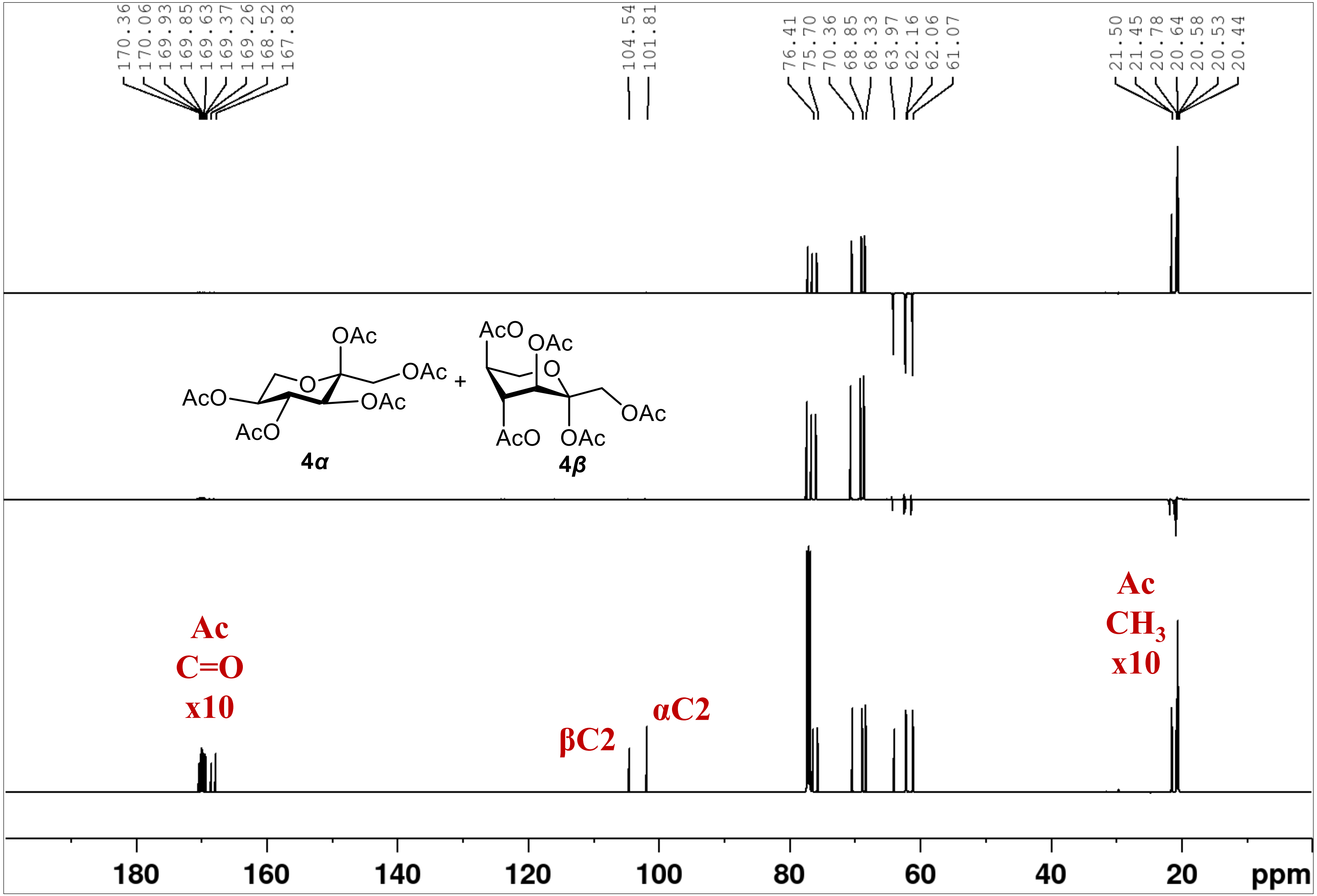


**Figure S20.** $^{13}$C, DEPT-90, and DEPT-135 NMR spectrum of compound **4*α*+4*β***.

# $^{1}H$-$^{1}H$ COSY

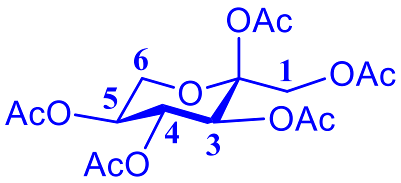


**4α**

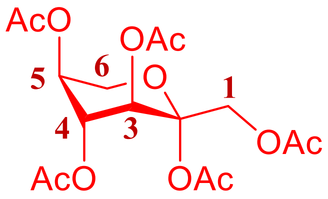


**4β**

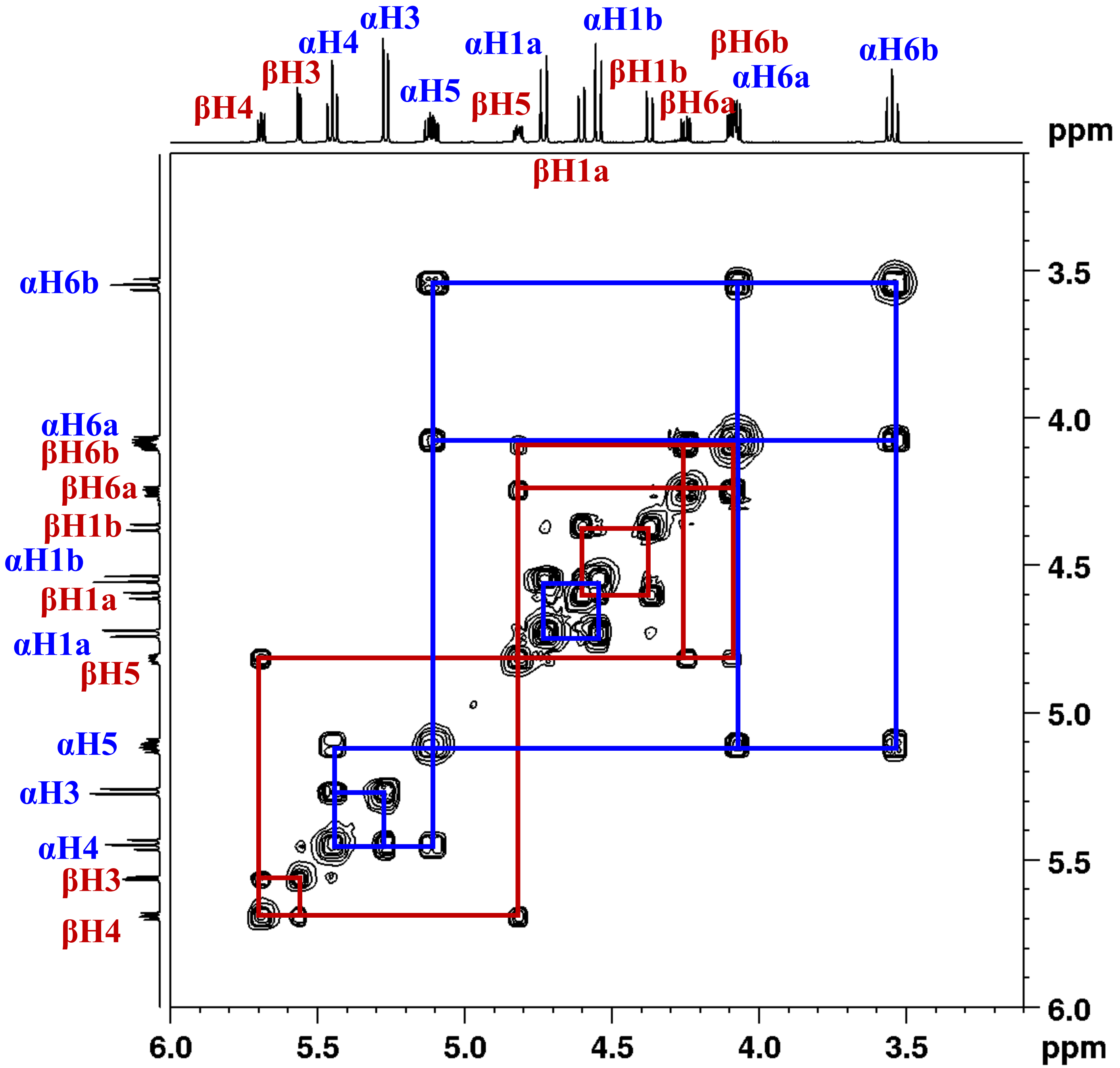


**Figure S21.** $^{1}H$–$^{1}H$ COSY NMR spectrum of compound **4*α*+4*β***.

# $^{13}C$-$^{1}H$ HSQC

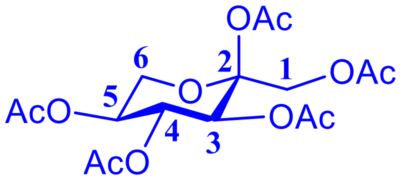


**4α**

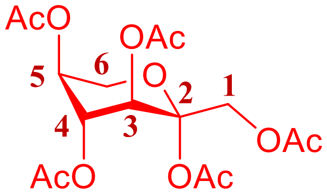


**4β**

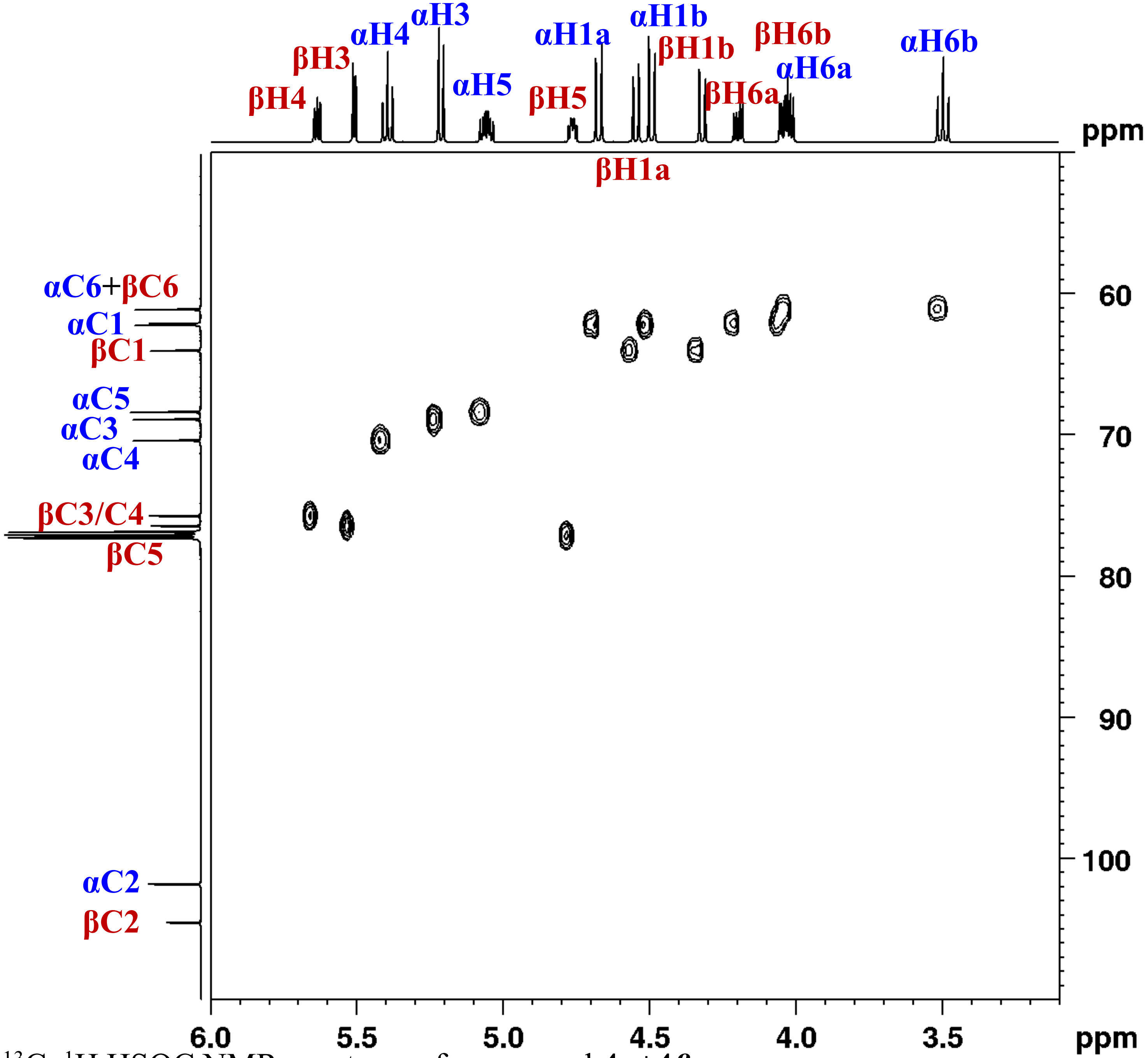


**Figure S22.** $^{13}C$–$^{1}H$ HSQC NMR spectrum of compound ***4α+4β***.

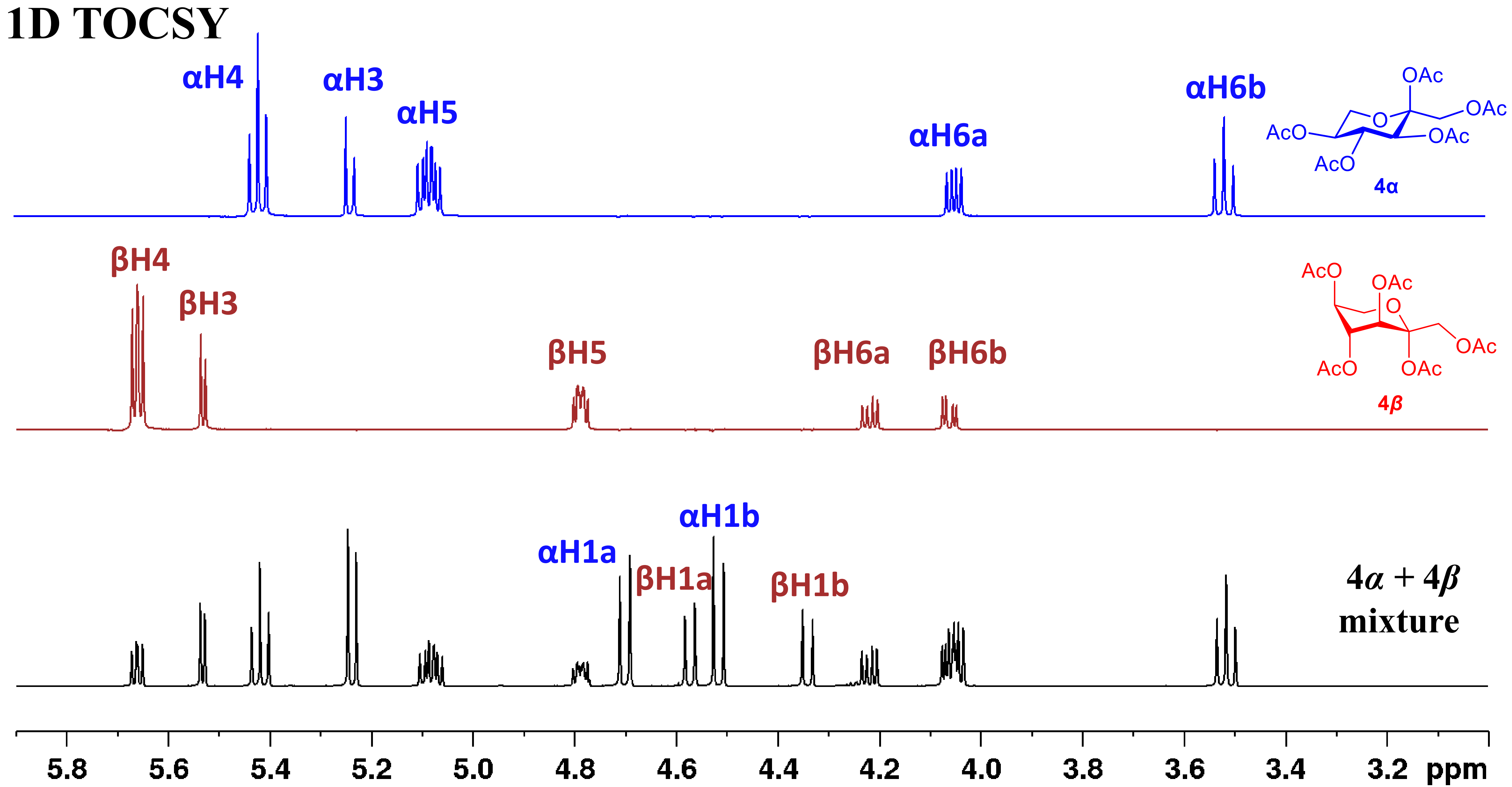


**Figure S23.** 1D TOCSY NMR spectrum of compound ***4α*+*4β***.

# IR Spectrum

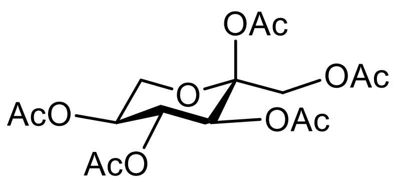


**4*α***

+

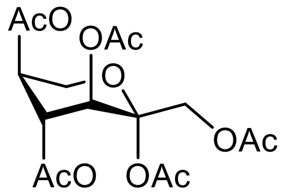


**4*β***

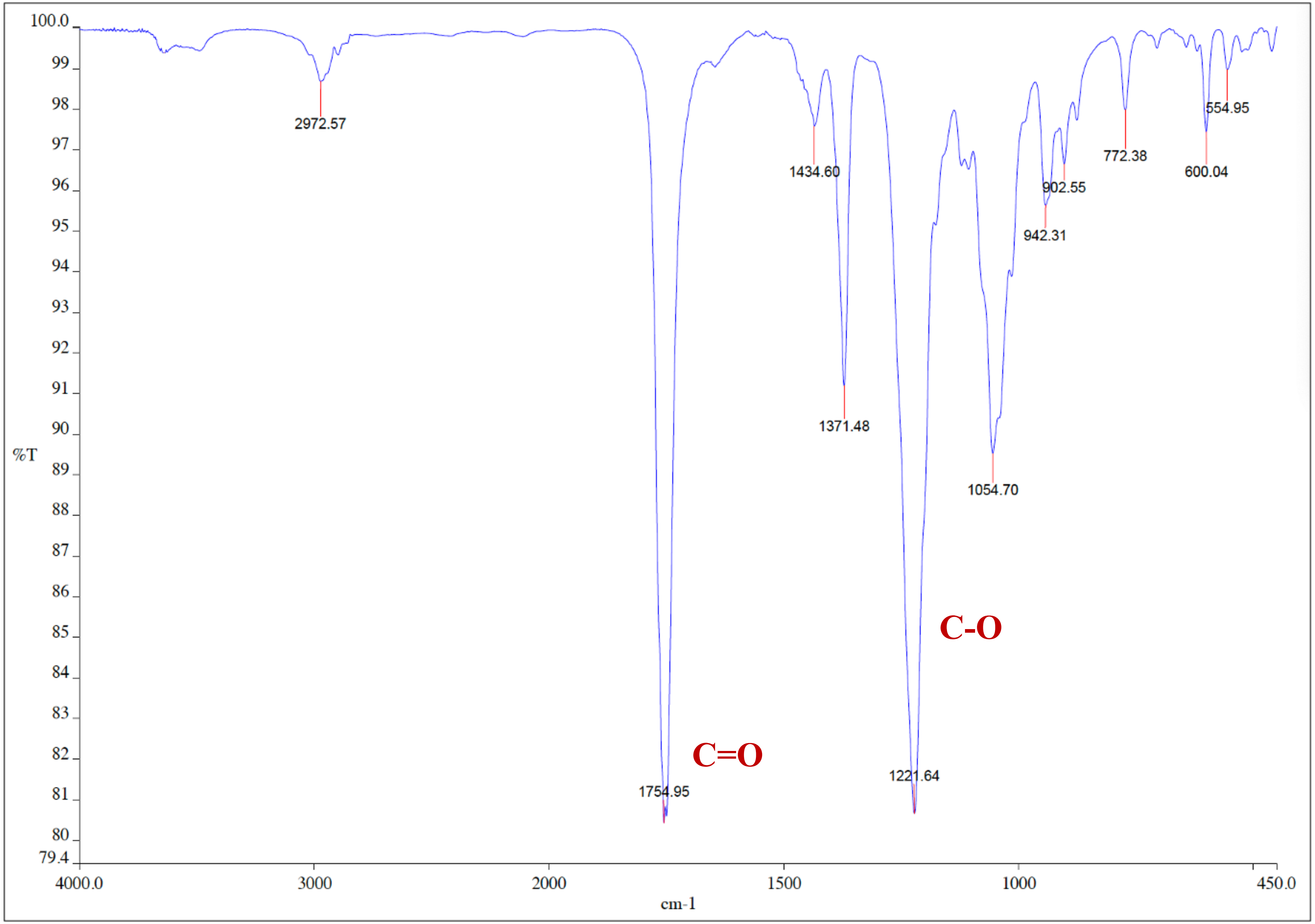


**Figure S24.** FT–IR spectrum of compound **4*α*+4*β***.

# HRMS–ESI

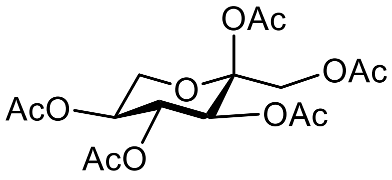


**4α**

+

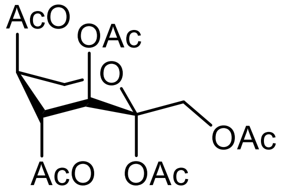


**4β**

**Calculated : 408.1500**

**Found : 408.1507**

**Mass Error : 1.72 ppm**

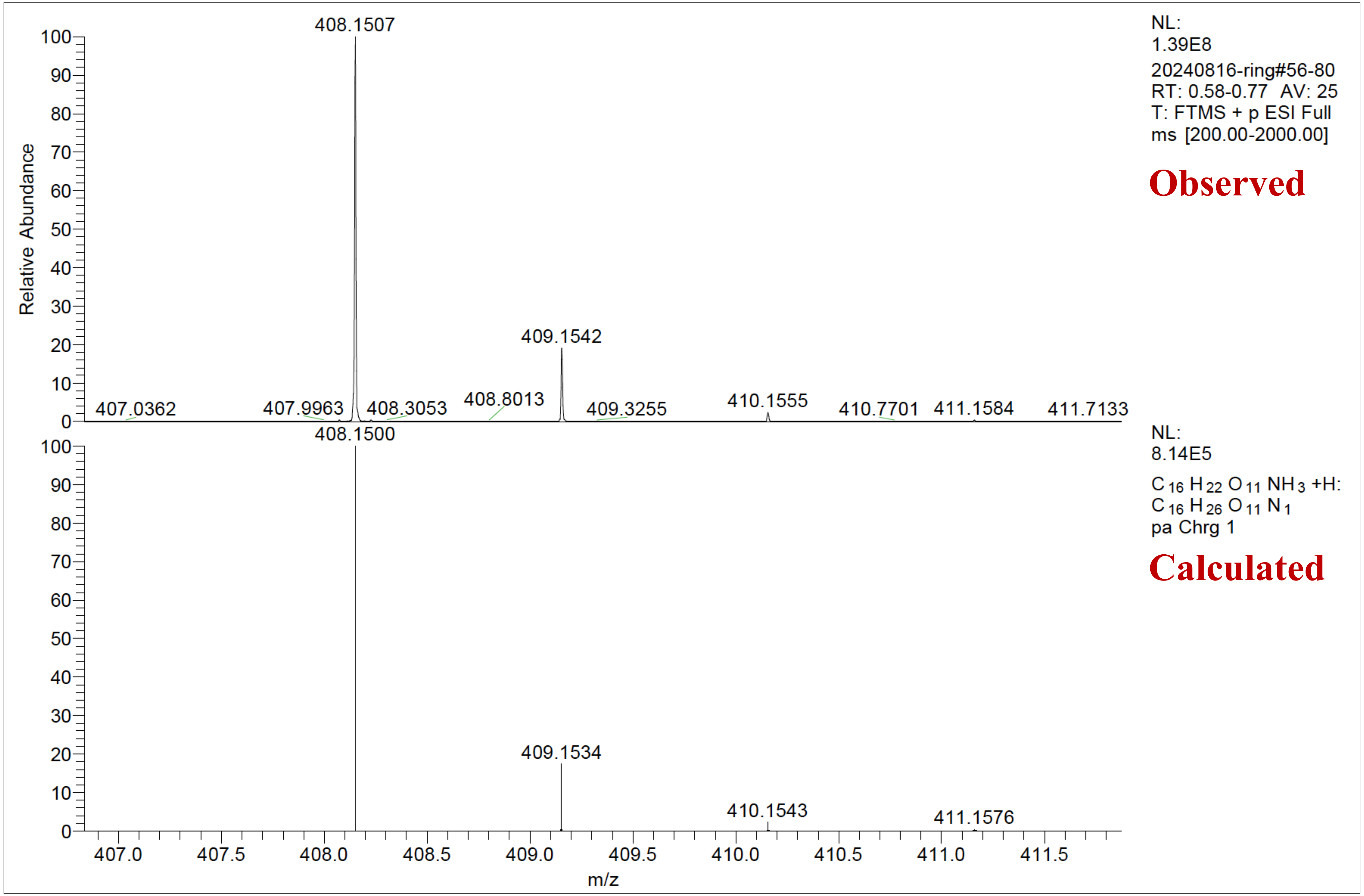


**Figure S25.** HRMS–ESI spectrum of compound **4*α*+4*β***.

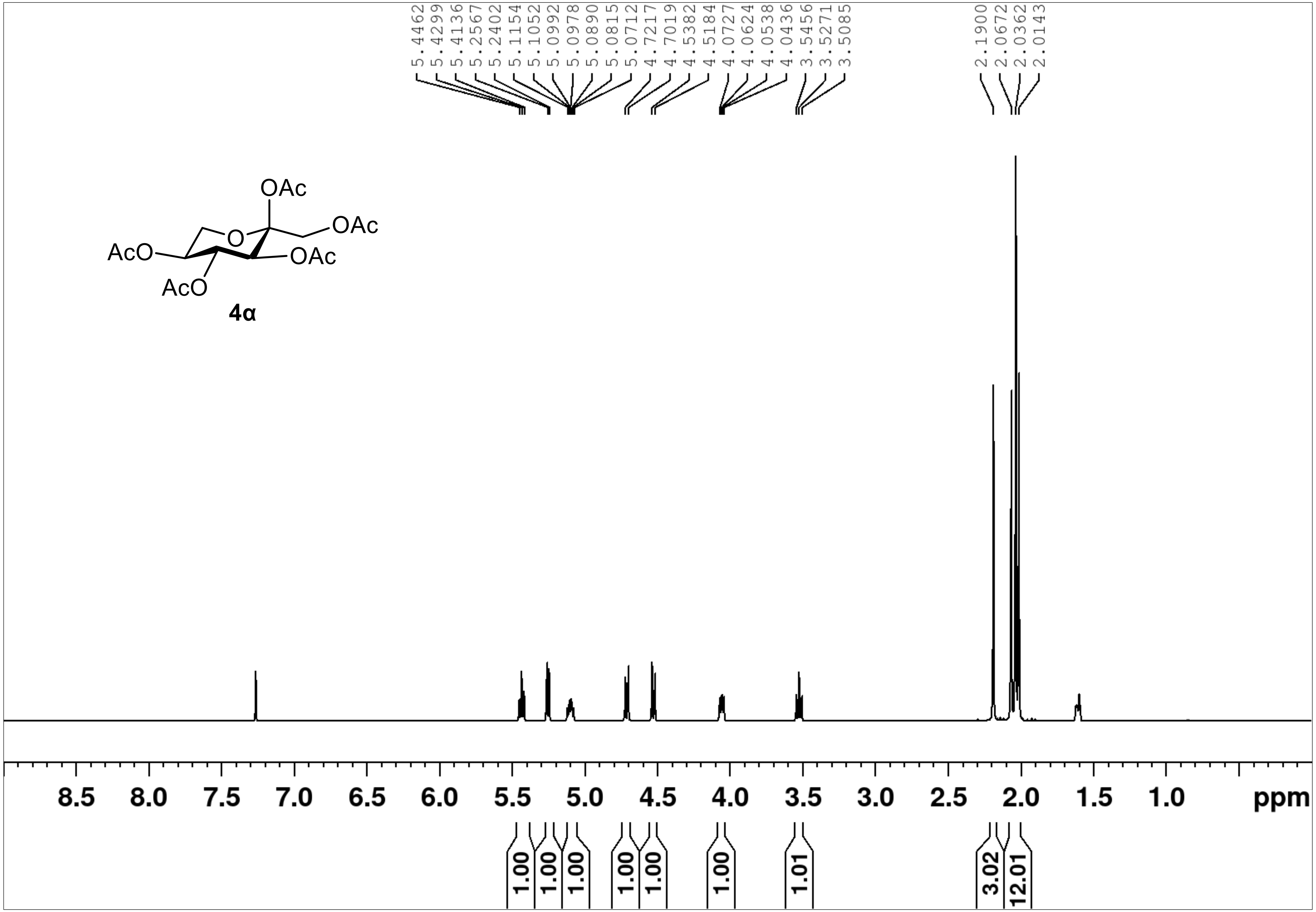


**Figure S26.** $^1$H NMR spectrum of compound **4*α***.

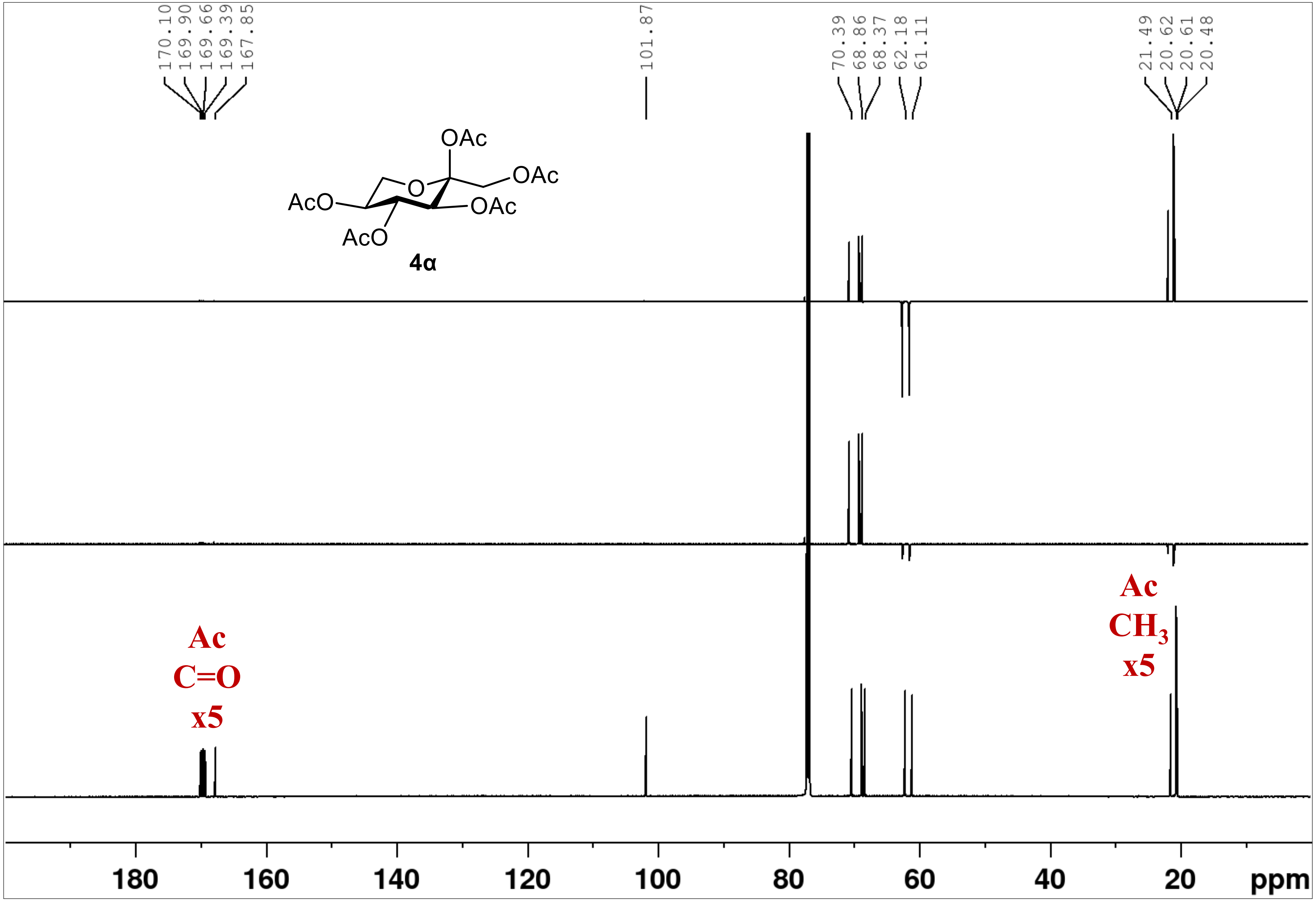


**Figure S27.** $^{13}C$, DEPT-90, and DEPT-135 NMR spectrum of compound ***4α***.

# $^1$H-$^1$H COSY

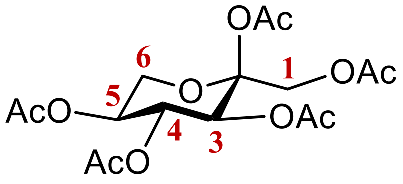


**4α**

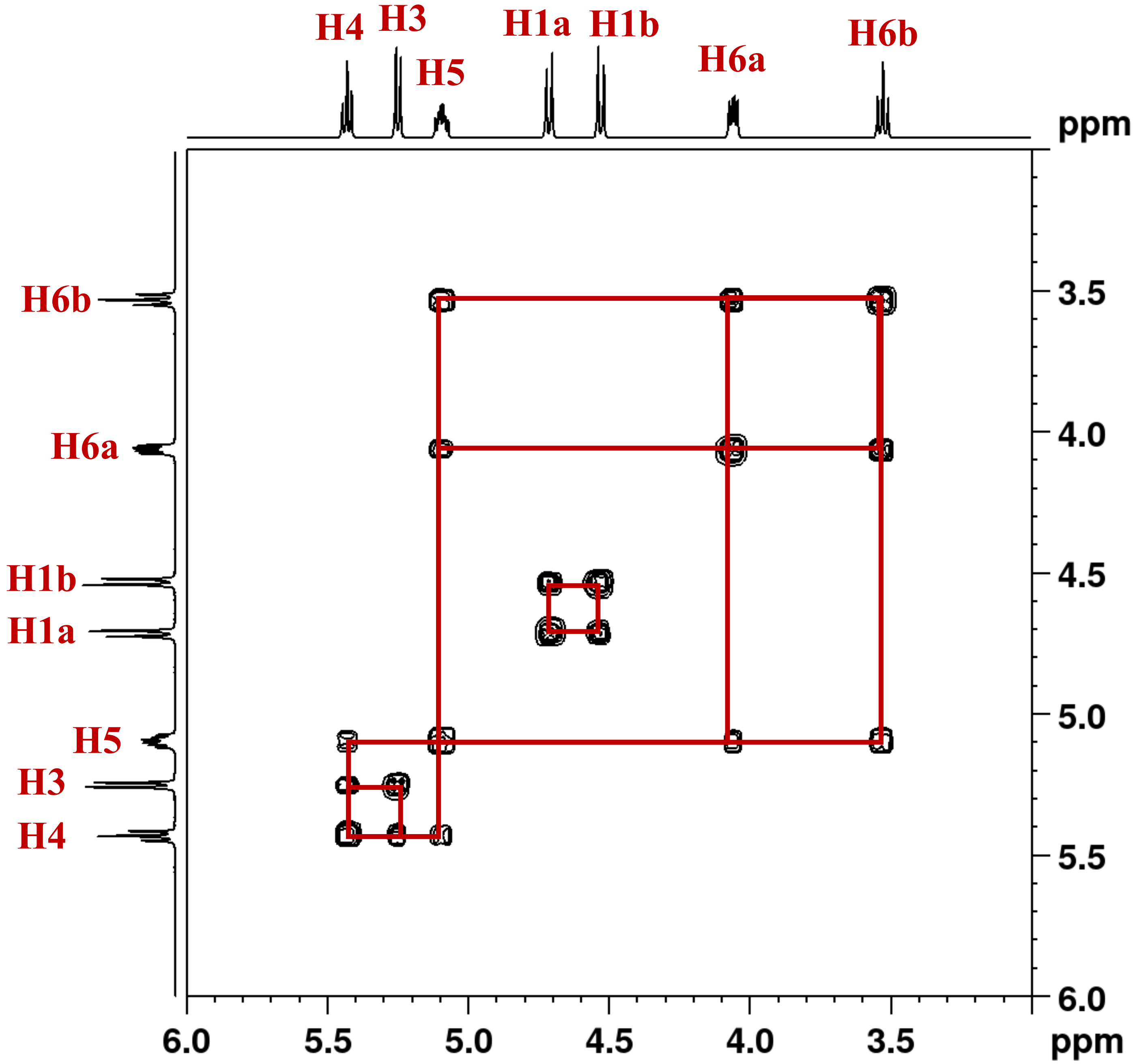


**Figure S28.** $^1$H–$^1$H COSY NMR spectrum of compound ***4α***.

# $^{13}C$-$^{1}H$ HSQC

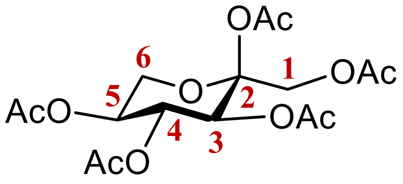


**4α**

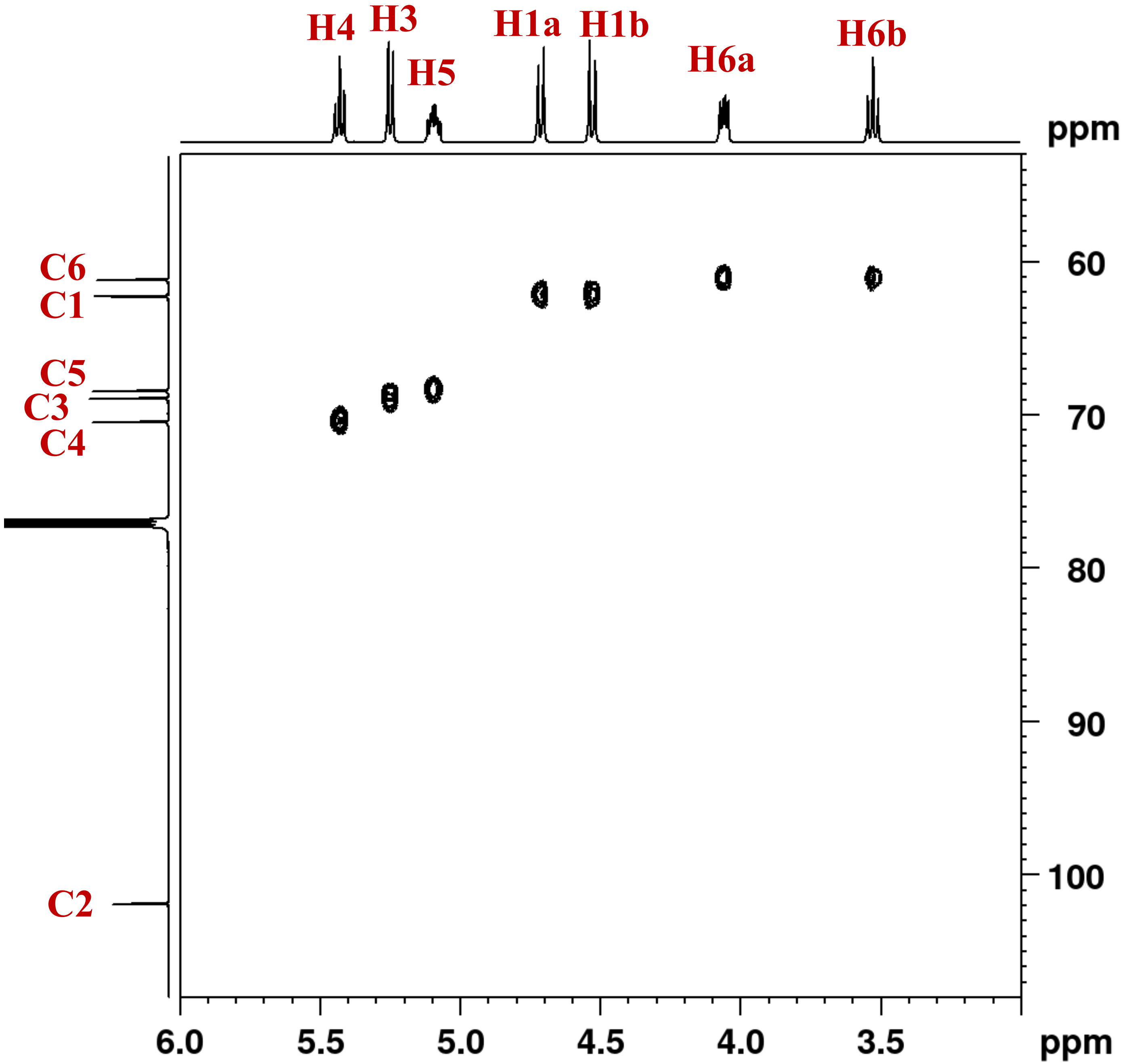


**Figure S29.** $^{13}C$–$^{1}H$ HSQC NMR spectrum of compound **4*α***.

# $^{1}H$-$^{1}H$ NOESY

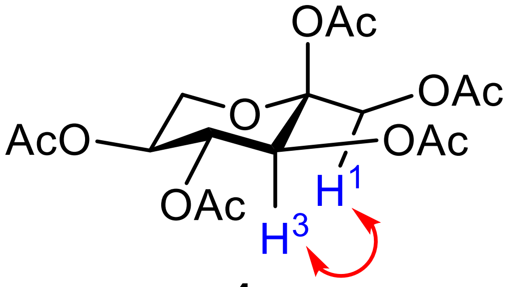


**4α**

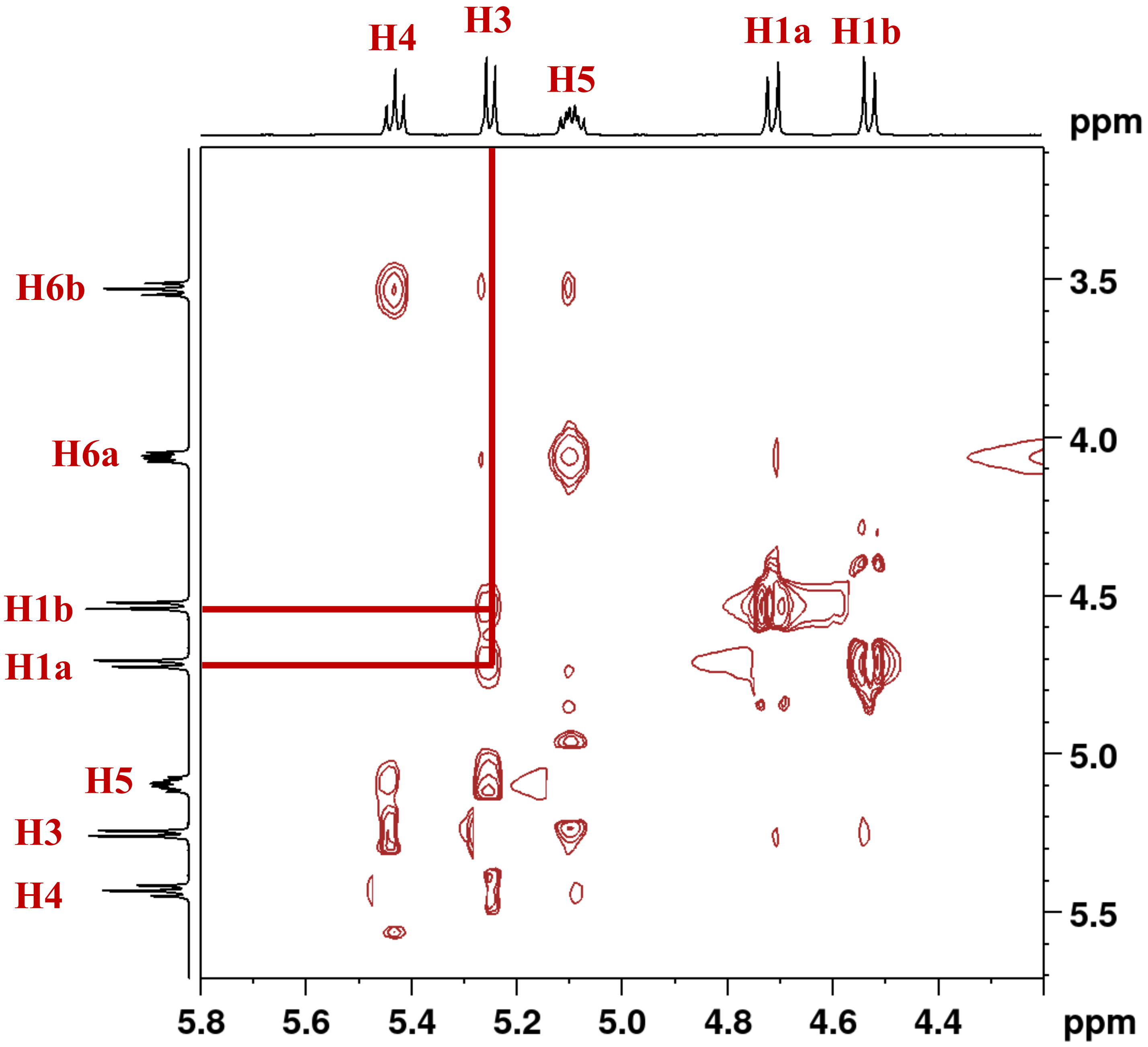


**Figure S30.** $^{1}H$–$^{1}H$ NOESY NMR spectrum of compound **4*α***.

# IR Spectrum

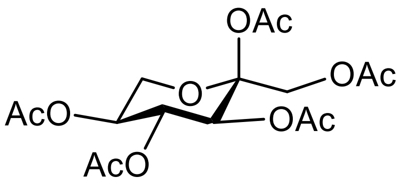


**4α**

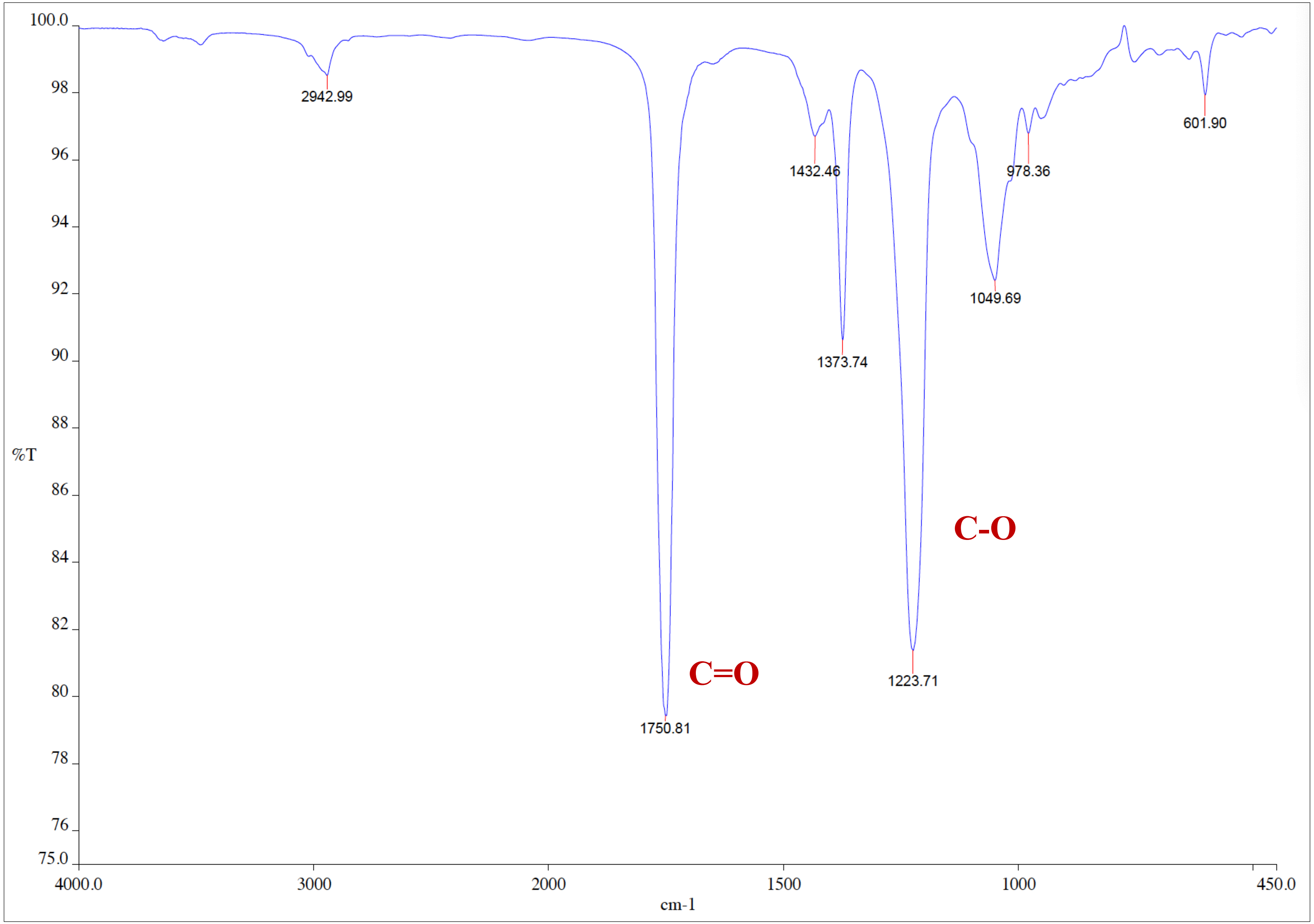


**Figure S31.** FT–IR spectrum of compound **4*α***.

# HRMS–ESI

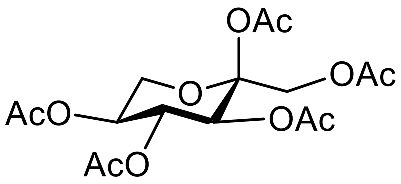


**4α**

**Calculated : 408.1500**

**Found : 408.1511**

**Mass Error : 2.70 ppm**

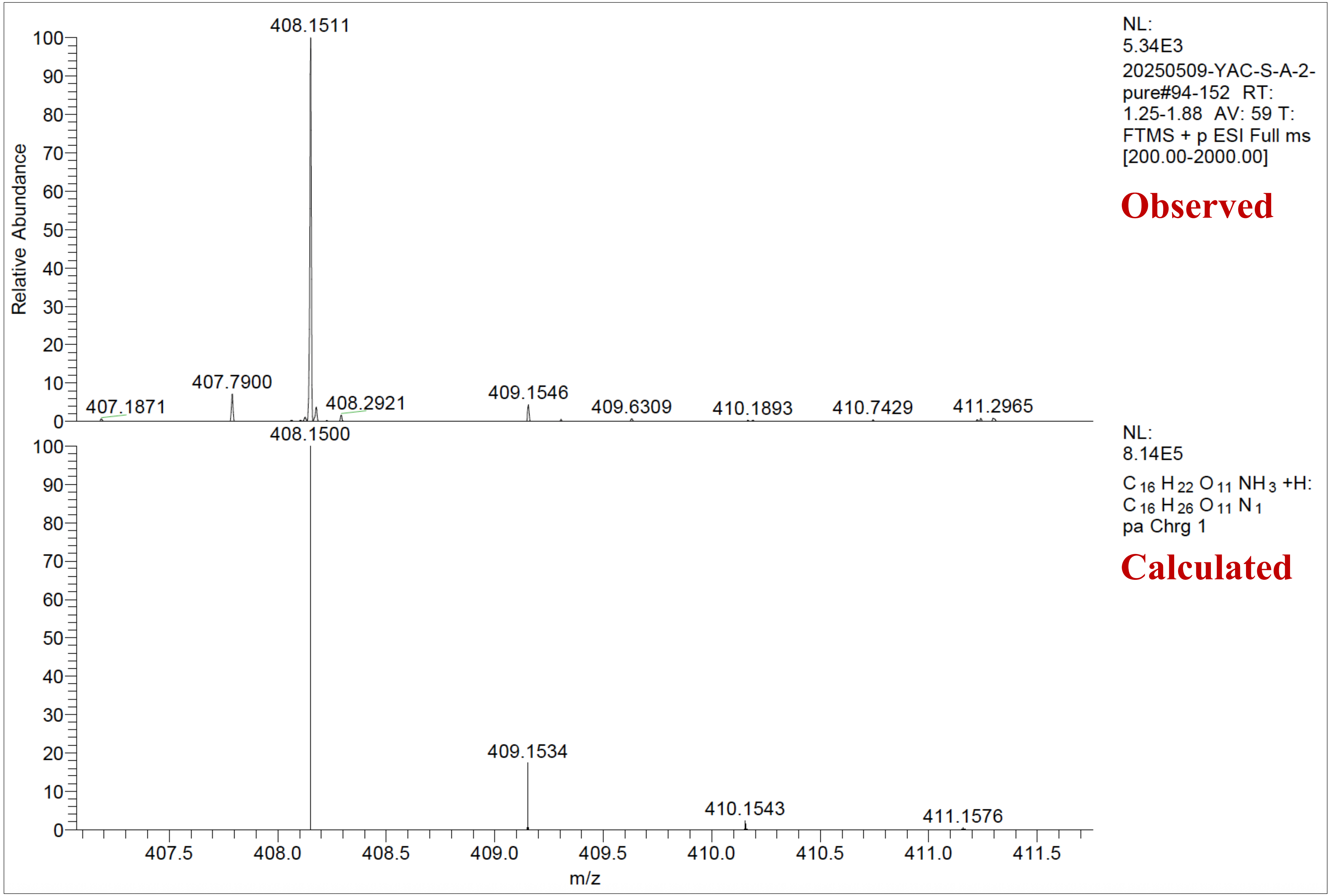


**Figure S32.** HRMS–ESI spectrum of compound ***4α***.

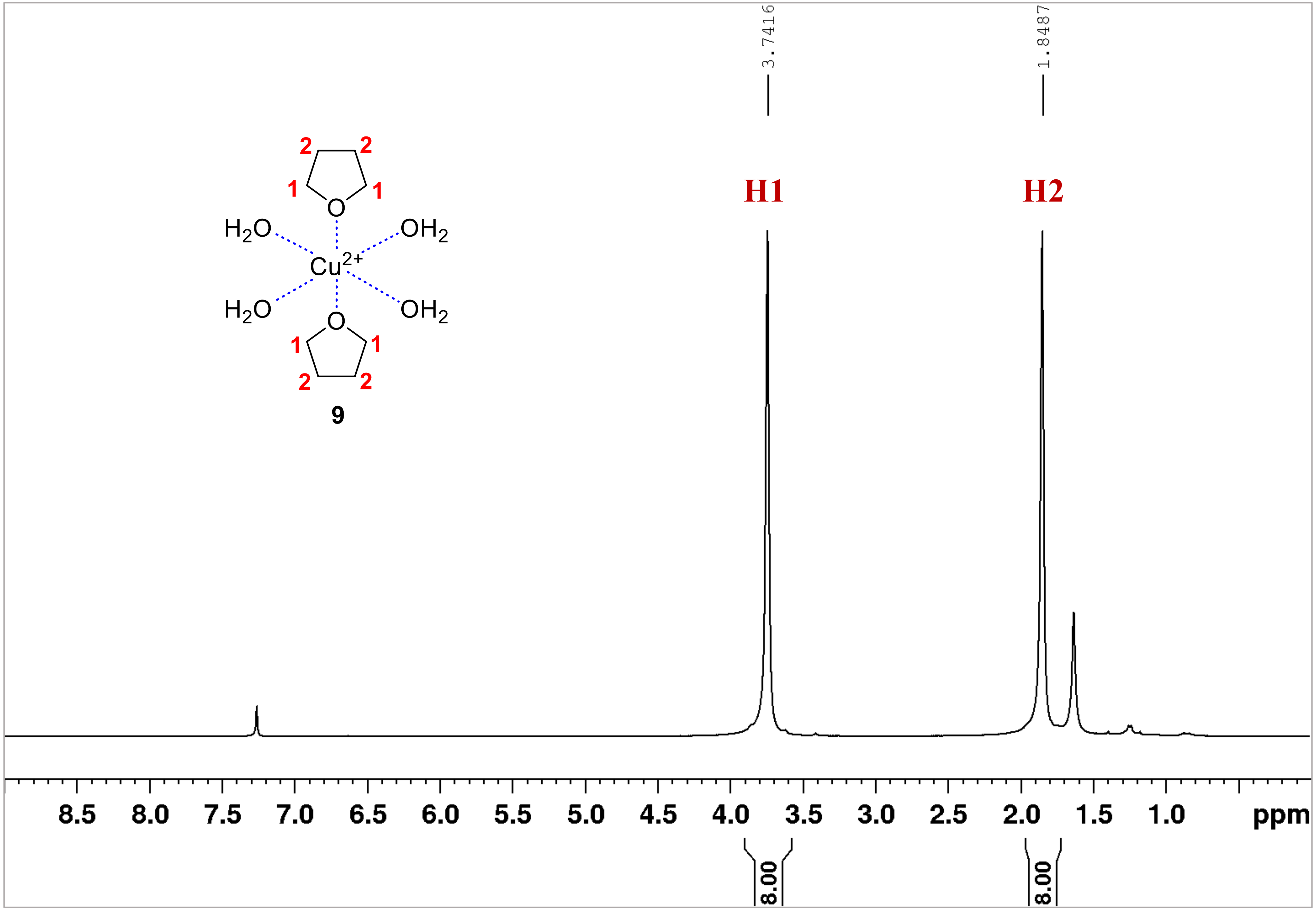


**Figure S33.** $^1$H NMR spectrum of compound **9**.

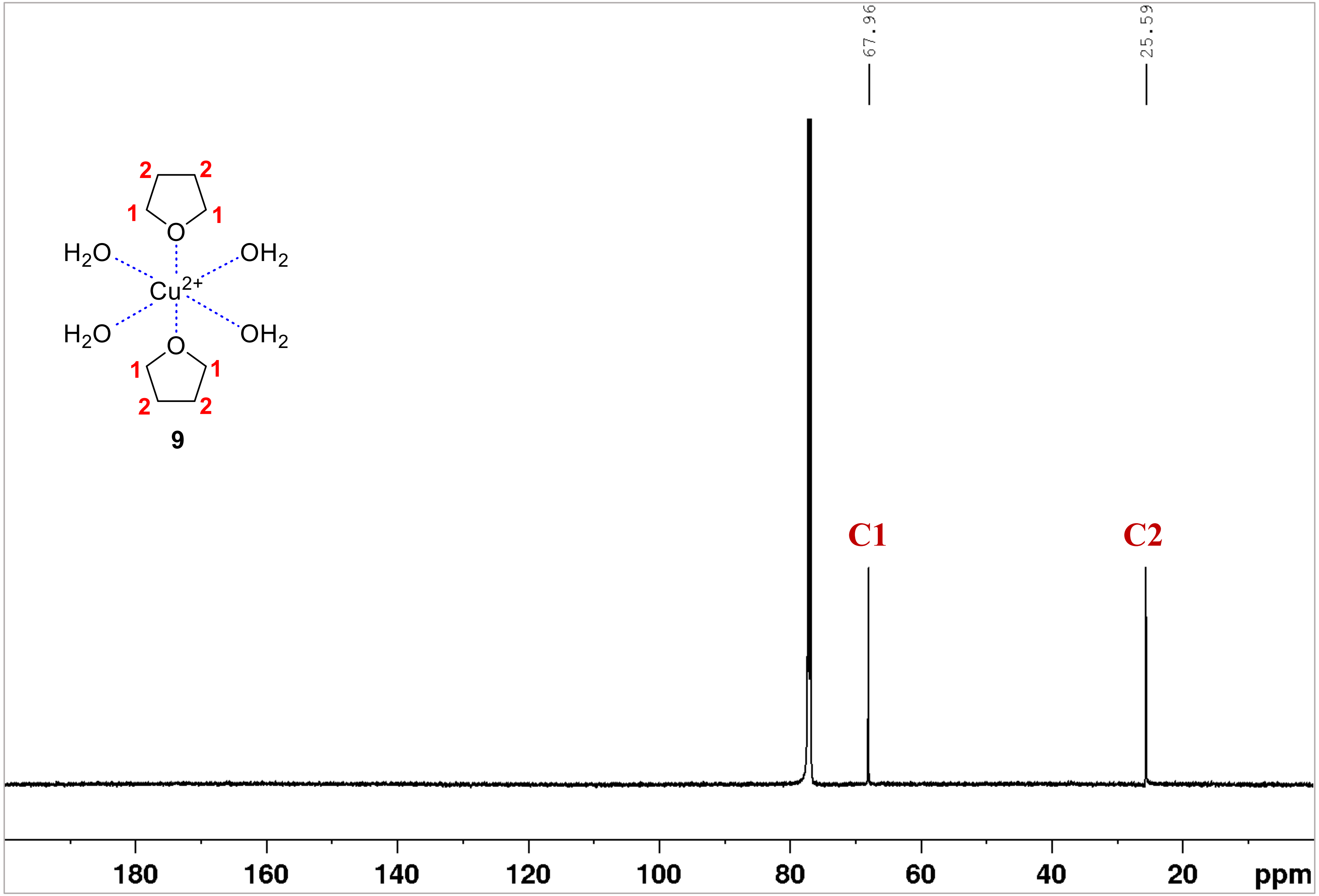


**Figure S34.** $^{13}$C NMR spectrum of compound **9**.
